# Ectopic expression of pectate lyase *Ptxt*PL1-27 in aspen affects leaf cuticle development

**DOI:** 10.1016/j.isci.2025.113963

**Published:** 2025-11-06

**Authors:** Ajaya K. Biswal, Alicja Banasiak, Josefina-Patricia Fernández-Moreno, Madhusree Mitra, Jesper Harholt, Marta Derba-Maceluch, Mateusz Majda, Sunita Kushwah, Vikash Kumar, Ilka Abreu, Pramod Sivan, Sivakumar Pattathil, Peter Immerzeel, András Gorzsás, Thomas Moritz, Michael G. Hahn, Henrik Vibe Scheller, Asaph Aharoni, Ewa J. Mellerowicz

**Affiliations:** 1 Umeå Plant Science Centre (UPSC), Department of Forest Genetics and Plant Physiology, Swedish University of Agricultural Sciences, 90183 Umeå, Sweden; 2 Department of Biochemistry and Molecular Biology, University of Georgia, Athens, GA 30602, USA; 3 Complex Carbohydrate Research Center, University of Georgia, 315 Riverbend Road, Athens, GA 30602, USA; 4 Department of Plant Developmental Biology, Faculty of Biological Sciences, University of Wrocław, Kanonia 6/8, 50-328 Wrocław, Poland; 5 Instituto de Biología Molecular y Celular de Plantas (CSIC-UPV), Ciudad Politécnica de la Innovación, Universidad Politécnica de Valencia, 46022 Valencia, Spain; 6 University of Copenhagen, Thorvaldsensvej 40, 1871 Frederiksberg C, Denmark; 7 The Mechanobiology Laboratory, Department of Plant Molecular Biology, University of Lausanne, 1015 Lausanne, Switzerland; 8 Department of Chemistry, Umeå University, 90187 Umeå, Sweden; 9 Joint BioEnergy Institute, Lawrence Berkeley National Laboratory, Berkeley, CA 94720, USA; 10 Department of Plant and Environmental Sciences, Weizmann Institute of Science, Rehovot 76100, Israel

**Keywords:** Plant Biology, Plant physiology, Plant development, Plant anatomy

## Abstract

Cuticle - a hydrophobic barrier of cutin and waxes covering the outer cell wall surface of plants - enables survival in terrestrial habitats. However, it is not understood how the hydrophobic cuticle precursors travel through the homogalacturonan-rich hydrophilic cell wall. To elucidate the role of homogalacturonan in cuticle development, we disrupted its integrity by overexpressing a pectate lyase, *Ptxt*PL1-27, in aspen. *Ptxt*PL1-27 had pleiotropic effects on shoot development, including the reduction of cuticle thickness and changes in cutin and wax composition, but the expression of cutin biosynthetic genes was little affected. Despite a reduction in homogalacturonan content in the leaves, labeling with the homogalacturonan-specific antibody JIM5 in the outer epidermal cell wall layer increased and displayed an altered pattern. Moreover, the ultrastructure of cell walls was changed concomitant with lipid accumulation. We propose that the disruption of homogalacturonan integrity affected the cutinsome-dependent transport and polymerization of cutin monomers in the cell wall.

## Highlights

• Overexpression of pectate lyase *Ptxt*PL1-27 in aspen disrupts homogalacturonan
• *Ptxt*PL1-27 reduces cuticle thickness and modifies its composition
• *Ptxt*PL1-27 alters cell wall ultrastructure and carbohydrate-lipid distribution
• The results suggest a key role for homogalacturonan in cuticle development

## Graphical abstract

**Figure:**
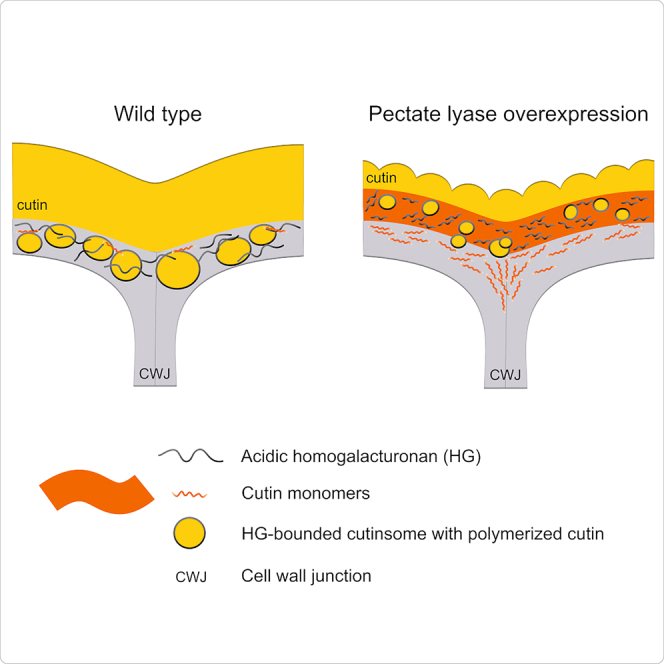

## Introduction

Plant leaf epidermal cells develop a layer of cuticle, forming a gas and water impermeable barrier that enables protection against biotic and abiotic stresses and regulation of gas exchange, which is of key importance for CO_2_ assimilation. The evolution of the cuticle was critical for plants to colonize terrestrial habitats, and it evolved along with stomata to allow gas exchange, which are capable of opening and closing according to environmental conditions and internal plant signals.^1^^,^^2^ The cuticle mostly comprises hydrophobic compounds, cutin, and waxes.^3^ Cutin is a branched polyester composed of glycerol acylated by C16 and C18 hydroxylated fatty acids (FAs) associated with minor, sometimes covalently linked, compounds, such as the phenolic compounds (cinnamic acid and flavonoids) or terpenes. Waxes are embedded in cutin (intra-cuticular) or surface-localized (extra-cuticular) and are composed of unpolymerized long-chain FAs, aldehydes, alcohols, alkenes, and alkyl esters. The structure of the cuticle considerably varies among species and developmental stages, but usually comprises three layers: an outermost layer of epicuticular waxes, underlain with cutin and waxes (cuticle proper), and finally, cutin and polysaccharides, which are in direct contact with the polysaccharides of the cell wall.^4^^,^^5^^,^^6^^,^^7^ The aliphatic building blocks of the cuticle are synthesized in plastids, modified in the endoplasmic reticulum, and finally transported through the cytoplasm to the apoplast, where they are polymerized.^1^^,^^2^^,^^3^ How the lipophilic waxes, cutin monomers, and oligomers are transported through the largely hydrophilic cell wall environment is still unclear. Some evidence points to a lipid transfer protein with a GPI anchor (LTPG) being involved in the transport.^8^ However, mutant phenotype analysis of another epidermis-specific lipid transport protein, LTP*2*, which was thought to be involved in the transport, suggested that it rather functions in maintaining the contact between cutin and cell wall.^9^ Other data point to spontaneously formed nanostructures known as cutinsomes as being involved in the transport.^10^ In the presence of polygalacturonic acid, the cutinsomes acquire an outer shell made of this polymer,^11^ and they condense hydroxy FAs, favoring their polymerization.^12^ Thus, they could play a dual role in cutin monomer transport and polymerization, complementing the enzyme-driven polymerization.^13^ Yet another hypothesis is that the transport could be mediated by extracellular vesicles containing lipophilic components, as such vesicles were observed in connection with suberin^14^ and lignin^15^ biosynthesis.

The role of cell wall polymers in the transport of cutin and wax components through the cell wall is very little studied. It is known that esterified homogalacturonan (HG), unbranched rhamnogalacturonan I (RGI), and crystalline cellulose are strongly associated with cuticle.^7^ Interestingly, when cutin polymerization in tomato was impaired by a mutation in *CUTIN SYNTHASE 1* (*CUS1*), the methylesterification of cutin-associated HG was affected, pointing to a link between cutin and HG biosynthesis and modification *in muro*. HG is an abundant primary cell wall component and the most abundant type of pectin in most cell types. It can be covalently connected to other cell wall polymers, forming supramolecular networks. One type of such networks comprises HG, RGI, rhamnogalacturonan II (RGII), arabinogalactan protein, and xylan.^16^ Another type includes HG interacting with an extensin LRX8 and a peptide RALF4.^17^ HG polymer is made up of up to 200 residues of (1,4)-linked α-D-galacturonic acid (GalA) that can be modified by *O*-acetylation at the *O*-2 or *O*-3 position and methylesterification at the C-6 position.^18^ The HG backbone is synthesized in the Golgi apparatus by the GAUT family,^19^ methylesterified by different methyl transferases, including COTTON GOLGI-RELATED (CGR)2 and CGR3,^20^ QUASIMODO2 (QUA2)^21^ and QUA3,^22^ and acetylated by acetyltransferase POWDERY MILDEW RESISTANT 5/TRICHOME BIREFRINGENCE-LIKE 44 (PMR5/TBL44).^23^ The acetylated and highly methylesterified HG is deposited in the cell wall by exocytosis, where it is de-esterified by pectin methylesterases (PMEs)^24^ and acetyl esterases such as an atypical TBL family member *At*TBL38,^25^ which renders it more susceptible to wall-localized endo-polygalacturonases (EPGs) and endo-pectate lyases (PELs). PELs (EC 4.2.2.2) cleave the α-1,4 glycosidic bond between GalA residues of partly de-esterified and de-acetylated HG by a β-elimination mechanism, forming unsaturated *oligo*galacturonides.^26^ They belong to the family of polysaccharide lyases 1 (PL1) (www.cazy.org), which also includes exo-pectate lyases (EC 4.2.2.9) and pectin lyases (EC 4.2.2.10). Plant HG-acting enzymes form multi-gene families whose members frequently show tissue specificity.^24^

HG metabolism plays a crucial role in plant development, and its perturbation usually induces severe developmental abnormalities.^21^^,^^27^^,^^28^^,^^29^^,^^30^^,^^31^^,^^32^ In the epidermis, HG metabolism is implicated in the regulation of cellular adhesion.^27^^,^^32^^,^^33^ It has also been proposed to be involved in the formation of a cuticular layer,^11^ but the dependence of cuticle formation on HG has not yet been formally demonstrated. Here, we disrupted HG integrity by ectopically overexpressing the PEL *Ptxt*PL1-27^34^ in hybrid aspen (*Populus tremula x tremuloides*). This PEL is highly expressed at the end of cell expansion in the wood-forming tissues, and we have previously observed that its overexpression affected wood chemistry and saccharification, increasing the solubility of matrix polysaccharides. Here we report the effects of *Ptxt*PL1-27 on leaf development. We observed pleiotropic effects of overexpressing *Ptxt*PL1-27 on leaves, including accelerated early development, increased gas exchange, impaired stomatal function, and substantially reduced cuticle thickness with altered cutin and wax composition in expanded and mature leaves. The ultrastructure of the cuticle and the underlying cell wall was altered with highly distorted HG epitope distribution and accumulation of lipids in the cell wall. These results provide evidence of pleiotropic PEL effects on leaf and cuticle development. Although the mechanism of the observed changes is likely complex, we propose that *Ptxt*PL1-27 effects on cuticle could be related to the disruption of cutinsome-mediated cutin monomer polymerization and transport through the cell wall.

## Results

### Overexpression of pectate lyase *Ptxt*PL1-27 induced major developmental changes in shoots and affected cuticle function

**Figure 1:**
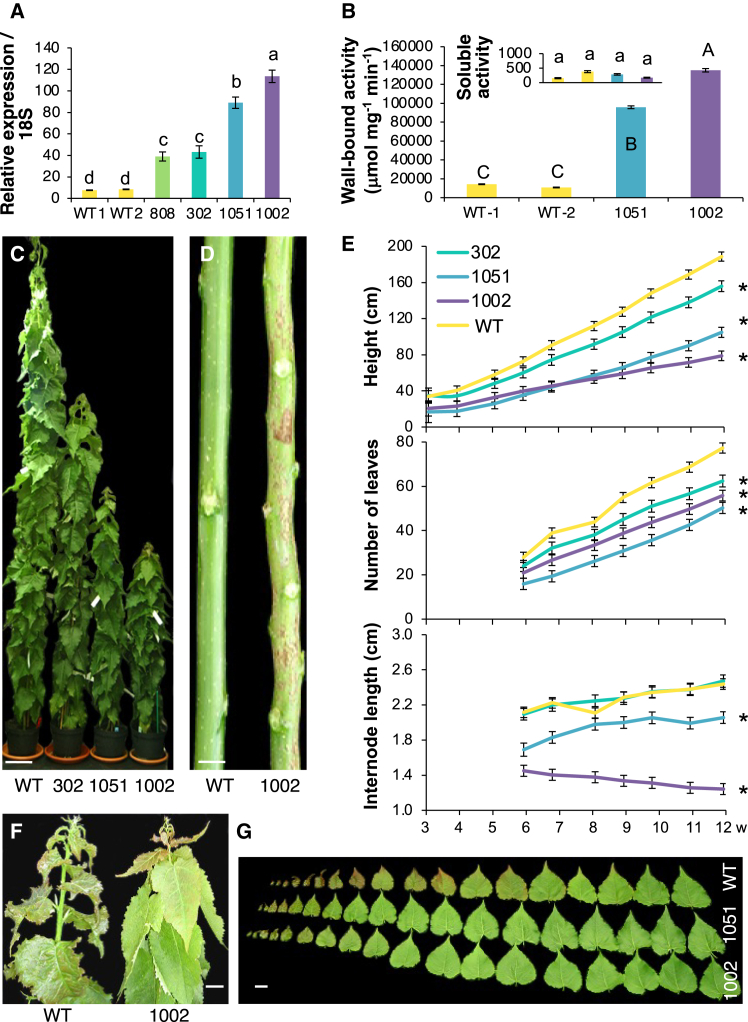
Overexpression of PEL affects shoot development (A) Relative *PtxtPL1-27* transcript level determined by RT-qPCR in apical developing shoot of WT and independent transgenic lines ectopically overexpressing *PtxtPL1-27*. (B) Pectate lyase activity in wall-bound protein fractions extracted from developing apical shoot tissues of WT and transgenic lines 1051 and 1002. The inset shows negligible activity in the soluble protein fraction. Data in A and B are means of two experiments, each with three biological replicates ±SD, WT-1 and WT-2 represent two sets of randomly selected wild-type trees; different letters indicate significant differences (Tukey test, *P* ≤ 5%). (C) Representative images showing the general plant appearance of the transgenic lines. (D) Defoliated stems showing extreme shortening of internodes and premature cork development in line 1002. (E) Dynamics of height, number of leaves, and internode length. Mean ± SE, for *N* between 8 and 12 trees. Weeks (w) refer to the duration of cultivation in the greenhouse. Asterisks indicate significantly different values compared to WT (Student test, *P* ≤ 5%) at the final date. (F and G) Representative images show the appearance of the apical part of the developing shoot in line 1002 and WT (F), and successive leaves in lines 1002, 1051, and WT (G). Note the accelerated leaf development in the transgenic lines. Scale bars = 10 cm (C), 1 cm (D), 2 cm (F), 5 cm (G).

*PtxtPL1-27* has previously been ectopically overexpressed in aspen.^34^ We selected two transgenic lines, i.e., 1002 and 1051, most strongly overexpressing *PtxtPL1-27* in developing shoots to study the effects of PEL on leaf development (Figure 1A). Most PEL activity was detected in the wall-bound protein fraction (Figure 1B), and it was 8- to 11-fold higher in the selected overexpressing lines compared to the wild type (WT). Overexpression of PEL reduced tree height by decreasing the internode length and number (Figures 1C–1E) and accelerated leaf expansion (Figures 1F and 1G). In the stem of the most affected line (1002), cork patches developed prematurely (Figure 1D). Also, the pavement cells in the abaxial and adaxial epidermis of the transgenic lines were more invaginated and larger than in WT at an equivalent developmental stage (Figure S1A). The anatomy of young expanded leaves was characterized by larger intercellular spaces, indicating reduced cellular adhesion, and larger cells in the mesophyll, leading to increased leaf thickness compared to WT (Figure S1B). Moreover, the size of the stomata in young expanded leaves increased (Figures S2A–S2F). When stomatal closure was induced by incubation with 5 mM CaCl_2_, the apertures closed with a concomitant decrease in stomatal size in WT, but the apertures remained open and the stomatal size was only slightly affected by closure in the transgenic lines. In agreement with the larger stomatal pores in the transgenic lines, the stomatal conductance, transpiration rates, and photosynthesis rates were higher in the mature leaves of transgenic lines than in WT (Table S1). In addition, the cuticle permeability was slightly affected in the expanding leaves of transgenic lines, as determined by the toluidine blue test^35^ (Figure S3A), and the water loss from young expanded detached transgenic leaves was more pronounced than in WT (Figure S3B).

### Pectate lyase *Ptxt*PL1-27 reduced pectin content and increased polysaccharide matrix extractability from leaves

**Figure 2:**
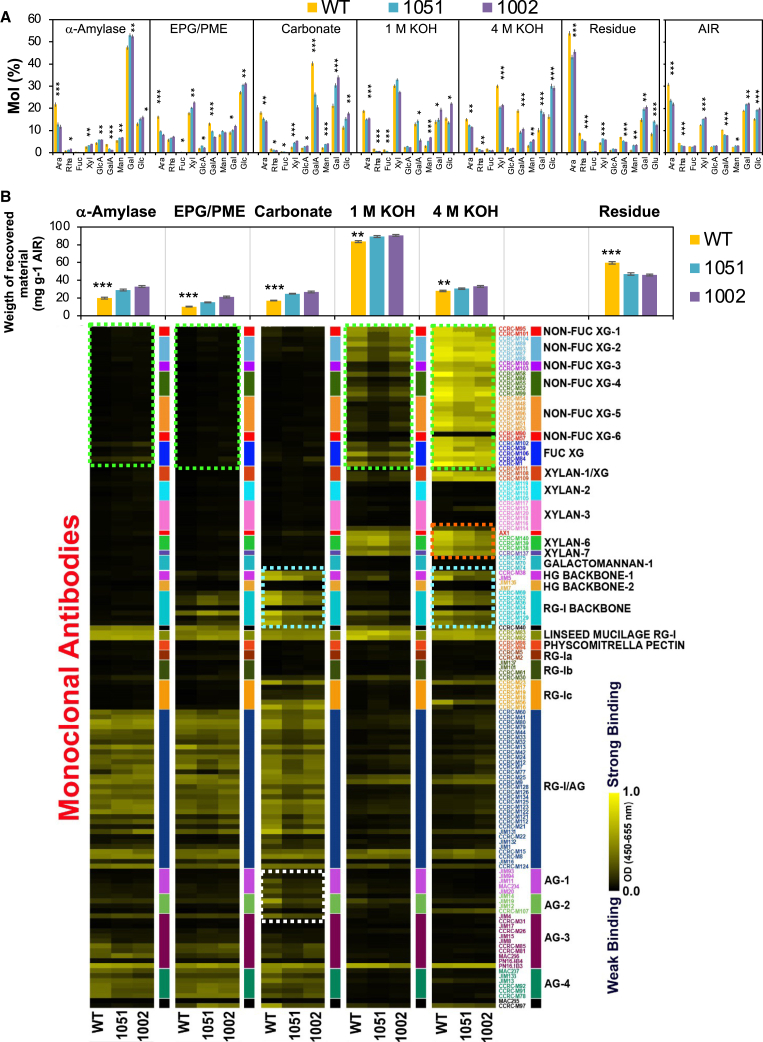
*Ptxt*PL1-27 induces major changes in sugar composition in cell walls in mature leaves (A) Changes in the sugar composition of different cell wall fractions and the alcohol-insoluble residue (AIR). The fractions correspond to extracts of AIR containing sequentially dissolved different cell wall polymers, as shown in B by immunodetection. (B) Glycome profiling of different cell wall fractions. Most prominent changes are marked by colored boxes: xyloglucan (green), xylan (orange), HG and RGI backbones (blue), and AG (white). Mean ± SE, *N* = 4. Asterisks indicate significant differences between WT and both transgenic lines (post-ANOVA contrast): ∗*P* ≤ 5%, ∗∗*P* ≤ 1%, ∗∗∗*P* ≤ 0.01%. See also Figure S4.

To characterize PEL induced changes in the cell walls of mature leaves, we analyzed the monosaccharide composition of matrix components in the alcohol-insoluble residue (AIR) that represents cell wall polymers with the possible contamination of starch, in successive extracts of AIR targeting different cell wall matrix polysaccharides, and in the residue (Figure 2A). This was followed by polysaccharide linkage analysis of these fractions (Tables 1, S2, and S3). Overexpression of PEL reduced the amount of HG in cell walls, as evidenced by decreased GalA content in the AIR and all cell wall fractions (Figure 2A) and decreased 4-GalA*p* content in the amylase fraction (containing water-soluble cell wall polymers), EPG/PME fraction (containing HG oligomers solubilized by EPG and PME), and carbonate fraction (targeting other pectins, mainly RGI) (Table 1). Additionally, we observed decreases in Ara and Rha in the AIR and Ara in every cell wall fraction (Figure 2A), along with a decrease in 4-Ara*p* or 5-Ara*f* in the amylase and EPG/PME fractions and 2,4-Ara*p* or 2,5-Ara*f* in the amylase fraction (Table 1). These results indicate decreased levels of arabinan in the transgenic lines in addition to decreased HG. Xyl content decreased in the 4 M KOH fraction (targeting tightly bound hemicelluloses) and slightly increased in the AIR, amylase, and pectic (EPG/PME and carbonate) cell wall fractions (Figure 2A). Linkage analysis (Table 1) indicated that most of the Xyl could be related to xylan/glucuronoxylan, as the xylan-related linkage content increased in the EPG/PME, carbonate, and 1 M KOH (including mostly loosely bound hemicelluloses) but decreased in the 4 M KOH fraction. This is indicative of increased xylan/glucuronoxylan extractability in the transgenic lines. Also, the solubility of some xyloglucan fractions in the transgenic lines seemed to increase, as evidenced by increased levels of 2-Xyl*p* and 2,4-Glc*p* in the carbonate fraction and their decreased levels in the 4 M KOH fraction of the transgenic lines compared to WT (Table 1). Gal content increased in the carbonate and in 1 M KOH and 4 M KOH fractions of the transgenic lines (Figure 2A). Linkage analysis indicated that both RGI galactan and arabinogalactan type II (AGII) could be responsible for this increase (Table 1). In addition, mannan (4-Man*p* or 2,3,6-Man*p*) increased in both hemicellulose (KOH) fractions of the transgenic lines compared to WT. Thus, the depletion of xylan in the 4 M KOH fraction of the transgenic lines was compensated by increased mannan and galactan.

**Table 1:** Polysaccharide linkages in different cell wall extracts significantly affected in lines overexpressing *PtxtPL1-27* compared to wild type (WT)

Linkage	Polysaccharide assignments	Amylase			EPG/PME			Carbonate			1 M KOH			4 M KOH		
		WT	1051	1002	WT	1051	1002	WT	1051	1002	WT	1051	1002	WT	1051	1002
t-Ara*f*	RGII/AGI/AGII/HX	3.1	**1.3**	**1.1**	9.9	**6.3**	**3.8**	3.8	**2.7**	**2.1**	6.8	**5.1**	5.6	0.06	**0.05**	**0.04**
4-Ara*p*/5-Ara*f*	Arabinan/RGI	7.2	**3.0**	**3.1**	5.9	**2.9**	**2.4**	5.3	4.1	3.8	1.9	1.6	1.3	7.3	6.0	5.6
2,4-Ara*p*/2,5-Ara*f*	Arabinan/RGI	4.4	**1.4**	**1.6**	–	–	–	2.4	2.6	2.7	–	–	–	1.9	1.6	1.5
2-Rha*p*	RGI	–	–	–	2.4	2.7	2.8	–	–	–	–	–	–	3.5	**2.6**	**1.5**
2,3-Rha*p*	RGI	0.3	0.4	0.5	0.4	0.4	0.5	0.3	**0.2**	0.3	–	–	–	0.1	**0.08**	**0.04**
2,4-Rha*p*	RGI	0.6	0.9	0.8	2.3	2.7	2.6	1.3	1.0	0.8	0.4	**0.2**	**0.2**	2.7	2.0	**1.2**
t-Fuc*p*	XG	0.2	0.2	0.2	0.2	**0.4**	**0.3**	0.6	0.5	0.4	0.7	0.4	**0.05**	0.9	0.6	0.5
t-Xyl*p*	XG	0.1	0.2	**0.6**	0.5	0.8	1.0	–	–	–	2.9	2.2	1.9	4.3	**2.8**	**2.3**
2-Xyl*p*	XG/RGII	–	–	–	–	–	–	–	–	–	2.4	2.0	1.7	3.8	**2.4**	**2.0**
4-Xyl*p*	HX	1.4	2.1	2.3	15.2	17.4	20.4	1.9	**3.4**	**4.8**	17.6	19.6	14.9	14.9	**9.6**	**8.0**
2,4-Xyl*p*	HX	0.5	0.8	0.9	0.2	**0.4**	**0.3**	0.5	**1.4**	1.0	2.1	**3.4**	**4.1**	–	–	–
4-Man*p*	HM	0.9	1.0	1.1	4.8	5.4	5.6	–	–	–	6.1	6.7	7.8	1.6	**2.8**	**3.9**
2,3,6-Man*p*	HM	–	–	–	–	–	–	–	–	–	0.7	**1.7**	**1.9**	–	–	–
2-GlcA*p*	RGII/HX	0.9	1.0	1.1	0.2	0.3	0.0	0.2	**0.3**	**0.3**	–	–	–	0.4	0.4	**0.3**
4-GlcA*p*	HX	1.8	2.3	2.1	0.4	0.3	0.0	0.4	**0.3**	**0.3**	–	–	–	–	–	–
2,4-GlcA*p*	HX	0.5	0.7	0.8	–	–	–	1.2	**2.1**	**2.5**	–	–	–	0.4	0.4	0.4
t-GalA*p*	HG/RGI/RGII	–	–	–	1.8	1.3	0.8	1.1	0.4	0.5	–	–	–	2.8	**1.6**	**1.9**
3-GalA*p*	Unknown	–	–	–	–	–	–	0.9	**0.5**	**0.4**	–	–	–	–	–	–
4-GalA*p*	HG/RGI	2.5	**1.3**	**0.9**	14.1	**9.8**	**8.2**	33.0	**15.7**	**12.9**	10.0	11.2	**4.3**	12.3	**8.0**	**8.2**
3,4-GalA*p*	RGI	0.8	**0.6**	**0.5**	–	–	–	6.5	**5.3**	**4.1**	–	–	–	0.9	0.5	0.6
5-Api*f*	AGII/RGII	0.2	**0.1**	**0.1**	–	–	–	0.6	0.3	0.4	–	–	–	0.8	0.5	**0.4**
t-Gal*p*	RGI/RGII/XG/HX	5.2	6.2	5.9	4.5	5.9	6.0	4.3	**7.4**	**8.8**	4.4	3.5	**3.1**	4.1	3.2	2.9
2-Gal*p*	XG/RGI	0.6	0.7	0.6	0.3	**0.5**	**0.8**	–	–	–	1.7	1.2	1.1	0.8	**0.2**	**0.3**
4-Gal*p*	AGI/RGI	1.4	1.8	1.9	1.0	1.7	1.5	2.7	**5.8**	**5.7**	1.1	1.0	0.9	4.8	**15.3**	**17.9**
6-Gal*p*	AGII/RGI/RGII	6.3	7.1	6.9	0.7	0.9	1.1	4.3	**7.3**	**8.4**	1.2	1.7	2.3	0.3	**2.4**	**2.2**
3,4-Gal*p*	AGI	2.6	2.9	2.7	–	–	–	–	–	–	0.7	**1.4**	**2.1**	0.5	**2.9**	**2.8**
3,6-Gal*p*	AGII/RGI	22.6	25.6	24.8	0.5	0.8	1.1	6.2	8.0	7.5	7.2	**8.9**	**10.1**	0.4	**1.6**	**1.3**
4,6-Gal*p*	AGI	–	–	–	–	–	–	–	–	–	–	–	–	0.2	**1.4**	**1.7**
3,4, 6-Gal*p*	AGII	3.6	4.1	4.4	–	–	–	0.9	**1.7**	**1.8**	–	–	–	–	–	–
t-Glc*p*	prob. starch	2.4	3.1	3.4	2.9	3.9	3.5	1.7	2.4	**2.9**	1.6	1.1	0.9	1.0	**6.0**	**7.8**
2-Glc*p*	unknown	1.0	1.2	1.2	0.3	**0.5**	**0.4**	0.4	0.6	0.5	–	–	–	0.1	**0.8**	**0.7**
4-Glc*p*	starch/GM	3.1	3.6	3.8	21.0	23.0	25.0	4.7	**7.4**	**8.9**	15.5	**12.9**	**12.6**	12.7	**8.8**	**8.4**
2,4-Glc*p*	prob. starch	0.6	0.7	0.8	–	–	–	0.6	0.9	1.0	0.2	**1.3**	**2.7**	0.4	**1.6**	**2.1**
3,4-Glc*p*	prob. starch	0.3	0.3	0.3	0.4	0.6	0.7	0.7	0.9	**1.1**	0.1	0.2	**2.6**	0.2	**1.1**	**2.2**
4,6-Glc*p*	XG/starch	0.4	0.5	0.5	1.8	2.0	2.2	1.2	**2.7**	**2.9**	3.3	2.8	2.5	4.5	**3.2**	**3.0**

Data are molecular percentage means of two replicates and are shown only for linkages that showed a significant difference between transgenic and WT samples (means in bold, *P* < 0.05, Dunnett test) for both transgenic lines in at least one fraction. Data on all linkages detected are shown in Tables S2 and S3. AGI and II – arabinogalactan I and II; GM – glucomannan; HX – heteroxylan; HM – heteromannan; RGI and II – rhamnogalacturonan I and II; XG – xyloglucan; - indicates linkages not detected.

Glycome profiling^36^ was subsequently applied to the same cell wall fractions to detect qualitative differences among polysaccharide epitopes in different cell wall extracts of transgenic and WT plants using 150 monoclonal antibodies (mAbs, Figure 2B). A decrease in signals from the HG and RGI backbones (turquoise boxes) was observed in the carbonate and 4 M KOH fractions, confirming the reduction in HG and RGI in the transgenic lines detected by linkage and sugar analyses. Statistical analysis of HG backbone signals (Figure S4A) shows their significant decrease in most extracts, further confirming the reduced HG content in cell walls of transgenic leaves. The signal from some arabinogalactan epitopes (white box in Figure 2B) was lower in the carbonate cell wall fractions of the transgenic lines than in WT. Decreases in xyloglucan epitopes in 1 M KOH and 4 M KOH fractions, where these epitopes were most abundant, but slight increases in the α-amylase and EPG/PME fractions (green boxes in Figures 2B and S4B) confirmed overall decreased xyloglucan content and increased xyloglucan extractability in the transgenic lines. Decreases in xylan epitopes in the 4 M KOH fraction (orange box in Figure 2B) were also observed, in agreement with decreased 4-Xyl*p* linkages in that fraction, indicating that tightly bound xylan as xyloglucan was reduced in the transgenic lines. Moreover, the total amount of polysaccharides extracted with all extractants was increased, whereas that in the residue was decreased in the transgenic lines based on analysis of reducing ends in the hydrolyzed fractions (histograms above the glycome profile in Figure 2B), indicating overall higher extractability of matrix polysaccharides compared to WT.

### Pectate lyase *Ptxt*PL1-27 affected the distribution of pectin and xyloglucan epitopes in the outer cell wall of epidermal cells

**Figure 3:**
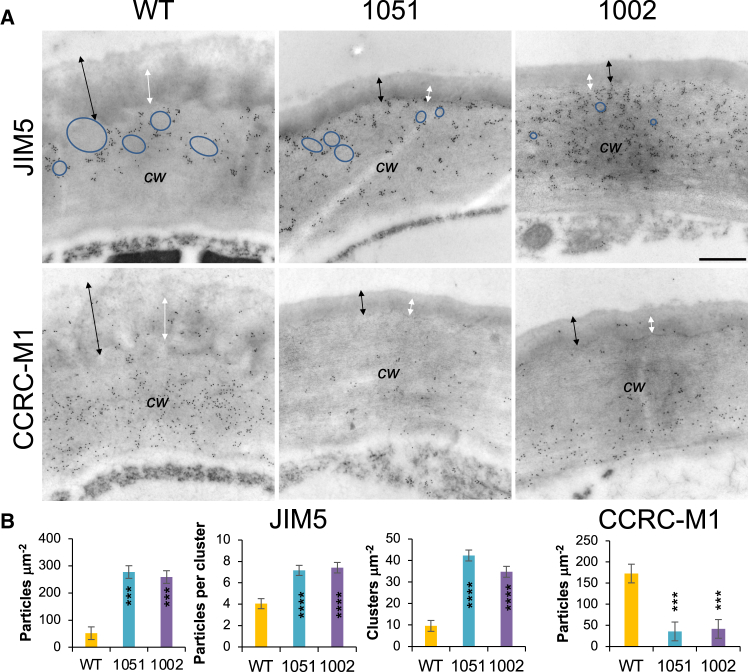
Overexpression of *PtxtPL1-2*7 affects the occurrence of homogalacturonan and xyloglucan epitopes in the outer cell wall of epidermal cells (A) Representative transmission electron micrographs show sections of the adaxial epidermis wall in the wild type (WT) and overexpressing lines 1051 and 1002 immunolabeled with JIM5 and CCRC-M1 antibodies, which recognize acidic homogalacturonan and fucosylated xyloglucan, respectively. Scale bars = 0.5 μm, *cw* – cell wall. Black arrows indicate the entire cuticle, whereas white arrows show the inner cuticle layer. Note that JIM5 labeling was found in clusters concentrated in the outer cell wall, with numbers markedly increased in the transgenic lines compared to WT, whereas the amount of label-free spaces (marked by blue ellipsoids) was reduced. In contrast, the CCRCM1 label was more evenly distributed in the inner cell wall layer and in small clusters in the inner cuticle layer (white arrows), and the density of gold particles was reduced in the transgenic lines. (B) Quantification of the JIM5 and CCRC-M1 signals. Mean ± SE, *N* = 12. Asterisks indicate significant differences from WT: ∗∗∗*P* ≤ 0.001; ∗∗∗∗*P* ≤ 0.0001; Dunnett test.

To establish how PEL affected the cell wall underlying the cuticle, we immunolocalized two of the most abundant matrix components of these walls, i.e., low methylesterified (acidic) HG and xyloglucan, using an immuno-gold technique (Figures 3A and 3B). Epitopes recognized by mAb JIM5 reacting with low methylesterified HG were mainly present in the outer cell wall layer directly adjacent to the cuticle layer (marked with a black arrow in Figures 3A and 3B). In WT samples, gold particles formed small clusters scattered around regions devoid of signal. In the transgenic lines, the regions devoid of label were much reduced, and more clusters of gold particles per area and more gold particles per cluster were observed. An overall density of JIM5 signals (number of gold particles per area) was over five times increased in the transgenic lines compared to WT.

In the case of fucosylated xyloglucan recognized by the CCRC-M1 antibody, the gold particles are more evenly distributed and mainly localized in the inner cell wall layer. The signals were drastically reduced in the transgenic lines compared to WT (Figures 3A and 3B). Thus, the JIM5 and CCRC-M1 signals exhibited opposite patterns of localization in the wall adjacent to the cuticle and opposite behavior in the transgenic lines. Some xyloglucan signals were also found in the inner layer of the cuticle, which was visibly darker (white arrows in Figure 3A). The presence of xyloglucan in this layer indicated that it was the cutin-polysaccharide layer, supporting a finding of covalent xyloglucan-cutin linkages.^37^

Decreased CCRC-M1 labeling in the epidermis of the transgenic lines was consistent with the reduced xyloglucan signals in 1 M KOH and 4 M KOH fractions detected by the glycome profiling of the leaves (Figure 2B). In contrast, increased JIM5 labeling of the outer epidermal walls of the transgenic lines was opposite to the overall reduction observed in all cell wall extracts of leaves of those lines (Figures 2B and S4A). This suggests that acidic HG in the outer epidermal cell wall has a specific conformation that differs from that in the bulk cell walls of the leaf.

### Pectate lyase *Ptxt*PL1-27 drastically decreased the thickness of the cuticle and reduced cutin deposition

**Figure 4:**
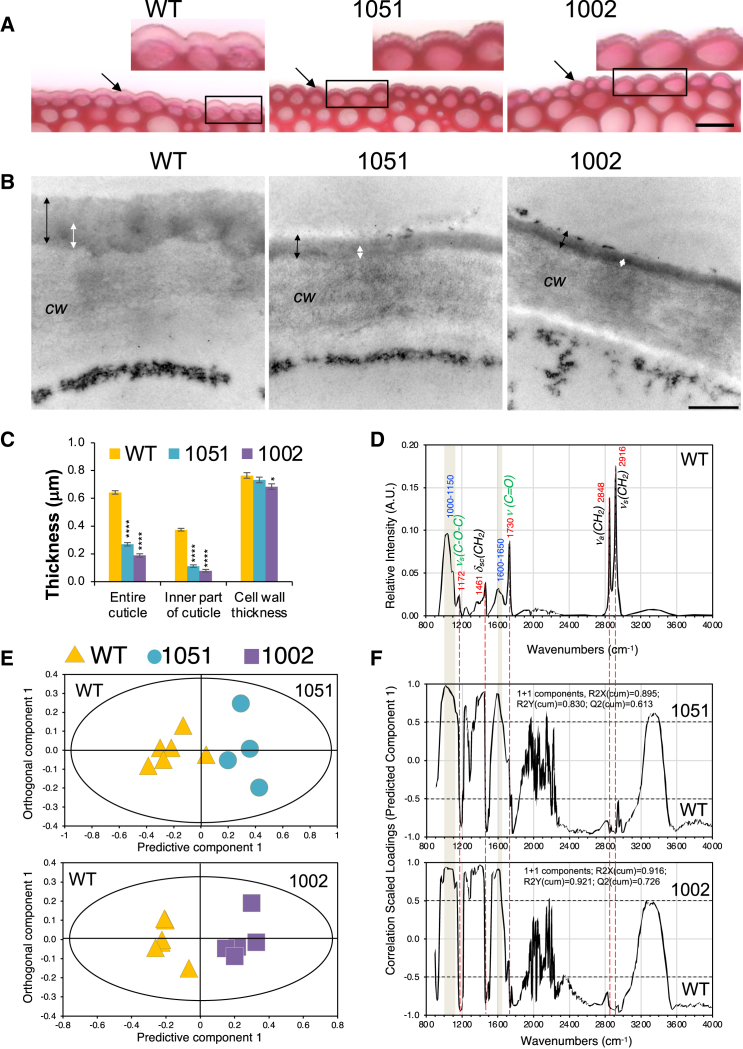
Overexpression of *PtxtPL1-2*7 affects the thickness and morphology of the cuticle of the adaxial epidermis (A) Representative images of fresh sections through the epidermis over the midrib stained with ruthenium red of wild type (WT) and transgenic lines 1051 and 1002. The arrows point toward the cuticle. Scale bars = 50 μm. The magnified areas show the granular appearance of the cuticle in the transgenic lines. (B) Representative transmission electron micrographs show sections of the epidermis outer wall in WT and overexpressing lines. Scale bars = 0.5 μm, *cw* – cell wall. Black arrows indicate the entire cuticle, whereas white arrows show the inner cuticular layer. Note that both the entire cuticle and its inner layer were substantially thinner in both transgenic lines, as also demonstrated by measurements (C). Mean ± SE, *N* = 9 (3 trees x 3 micrographs). Asterisks indicate significant differences from WT based on the post-ANOVA Dunnet test: ∗*P* ≤ 5%, ∗∗∗∗*P* ≤ 0.01%. (D–F) Overexpression of *PtxtPL1-2*7 affects the ATR-FTIR spectra of the epidermis. (D) Typical spectrum of cuticle in WT aspen. (E) OPLS-DA scores plots show the separation of the transgenic and WT samples. (F) Corresponding correlation-scaled OPLS-DA loading plots show spectral features contributing to the separation. Bands increased in the transgenic lines are shown in blue font, whereas bands reduced in the transgenic lines are shown in red font. Vibrations shown in black font are characteristic of cutin and waxes; vibrations shown in green font are characteristic for cutin only. Note the increased proportional intensities of carbohydrate-related bands (1000-1150 cm^−1^) and the –C=C– signal (1600-1650 cm^−1^) and the decreased proportional intensities of wax, cutin, and cutin-related signals in the transgenic lines.

We subsequently analyzed the cuticle of the transgenic lines through light and electron microscopy. Light microscopy images of fresh sections through the adaxial epidermis revealed significant reductions in the cuticle thickness and changes in the cuticle morphology in the transgenic lines (Figure 4A). Whereas in WT, the outer cuticle surface was relatively smooth, the surface in the transgenic lines contained small spherical structures (insets in Figure 4A). Transmission electron microscopy revealed that the entire cuticle and its inner darker layer (cuticle-carbohydrate layer) had a drastically reduced thickness in the transgenic lines, whereas the outer primary cell wall thickness was slightly reduced in line 1002 (Figures 4B and 4C).

We next used attenuated total reflectance Fourier transform infrared (ATR-FTIR) spectroscopy to determine the chemical nature of surface localized compounds affected in the adaxial epidermis cuticle of the transgenic lines. Two prominent peaks at 2916 and 2848 cm^−1^ and a smaller peak at 1461 cm^−1^ were observed (black font, Figure 4D), which are characteristic of fatty compounds in waxes and cutin.^38^ These signals significantly contributed to the separation between the transgenic lines and WT in the OPLS-DA analysis, with decreased intensities in the transgenic lines (Figures 4E and 4F). Two other distinct peaks at 1730 and 1172 cm^−1^ (green font, Figure 4D), characteristic of cutin,^38^ were also decreased in the transgenic lines. In contrast, a broad peak centered around 1100 cm^−1^ increased, most likely corresponding to higher proportions of polysaccharides.^39^ These results indicate that the transgenic lines had less cutin at the surface of the epidermis compared to WT. Signals of the broad peak between 1600 and 1650 cm^−1^ corresponding to C=C vibrations also increased in the transgenic lines. These signals can originate from aromatic C=C. In this case, however, stretching vibrations of the aromatic ring should also give rise to a band at 1500 cm^−1^, which should follow the band at 1600 cm^−1^. Since this was not the case, it is more likely that these signals are coming from non-aromatic C=C, such as a C=C bond generated by PEL in HG. Overall, the ATR-FTIR results provided evidence of a reduced cuticle in the transgenic lines and supported the degradation of HG by PEL.

### Overexpression of pectate lyase *Ptxt*PL1-27 affected the cutin and wax composition

**Figure 5:**
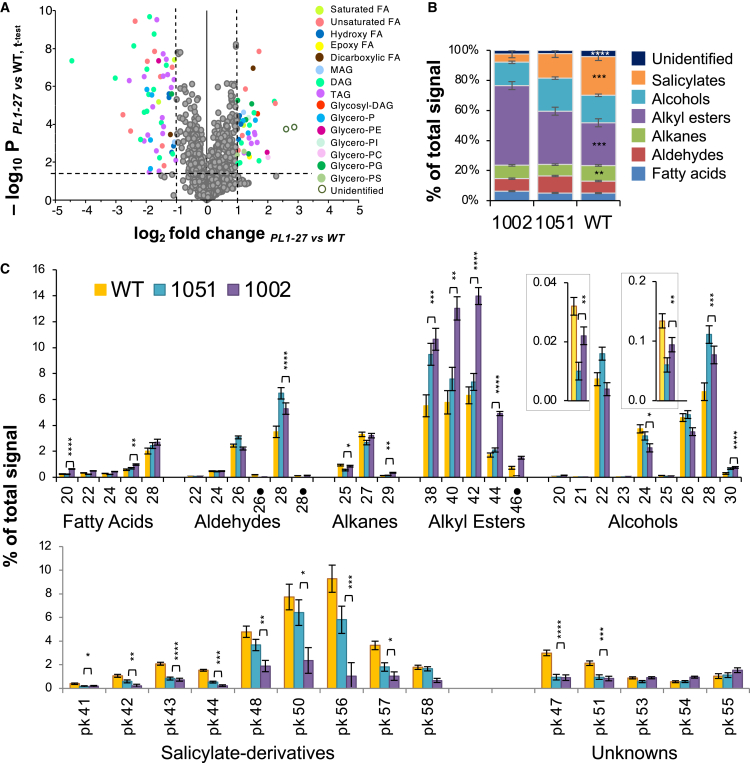
Overexpression of *PtxtPL1-2*7 affects the composition of cutin and cuticular wax (A) Volcano plot shows changes in cutin depolymerization products in the transgenic lines. Each colored dot represents a compound significantly changed in the transgenic lines (*P*_t test_ ≤ 0.05 and log_2_|fold change| ≥ 1), with different colors show different lipid categories: FA – fatty acid; P – phosphate; MAG – monoacylglycerol; DAG – diacylglycerol; TAG – triacylglycerol; PE – phosphoethanolamine; PI – phosphoinositol; PC – phosphocholine; PG – phosphoglycerol; PS – phosphoserine. The individual compounds are shown in Figure S4. (B) Changes in the content of major groups of wax compounds between the wild type (WT) and two overexpressing lines (1051 and 1002). (C) Changes in the relative content of individual wax compounds. Identified wax compounds have their chain length shown on the *x*-axis, whereas salicylate-derived polyphenols and unknown compounds are listed by their consecutive peak number. Black dot (●) indicates aldehyde derivatives. MS data for all compounds are listed in Table S6. Data in B and C are means ± SE, *N* = 5 biological replicates. Asterisks show compounds that differed between WT and the transgenic lines (post-ANOVA contrast) ∗*P* ≤ 5%, ∗∗*P* ≤ 1%, ∗∗∗*P* ≤ 0.1%, ∗∗∗∗*P* ≤ 0.1%).

To examine the effect of PEL on cutin composition, the cuticles of mature leaves were isolated, partially de-waxed by brief chloroform treatment, depolymerized, and then analyzed for their depolymerization products using a non-targeted metabolomics approach. A total of 936 compounds were identified by LC-MS analysis (Table S4), out of which 92 significantly differed in abundance between the transgenic and WT plants, and these compounds were putatively identified based on their mass (Table S5) and classified into 15 types of lipids (Figure 5A). Analysis of the relative abundances of these compounds (Figure S4) showed that the contents of some typical cutin monomers, e.g., C16 and C18 hydroxy FAs^1^^,^^3^ decreased in the transgenic lines. In contrast, saturated hydroxy FAs with longer chain lengths (C24-C30), which could represent suberin monomers,^3^ increased (Figure S5). Long mono- and polyunsaturated fatty acids (MUFAs and PUFAs) with chain lengths of C27-C30, which are reported components of cutin in some species,^40^ decreased in abundance, whereas those with even longer chain lengths (C31-C38) increased. Monoacylglycerol (MAG) 18:1 and smaller diacylglycerols (DAGs) increased in the transgenic lines, whereas larger DAGs and smaller triacylglycerols (TAGs) decreased. Thus, PEL induced some shifts in cuticle composition.

To determine whether PEL also affected extractable wax compounds, chloroform extracts of leaves were analyzed by gas chromatography-mass spectrometry (GC-MS)^41^ and 28 wax compounds, classified as FAs, aldehydes, alkanes, alcohols, and alkyl esters, were identified (Table S6). The wax coverage was at an average of 1.11 ± 0.15 μg cm^−2^ (mean ± SE) for both WT and transgenic plants, and it was not affected by PEL. However, the composition of the extracted wax showed prominent changes that followed the transgene expression levels: a decrease in salicylate compounds (including tremuloidin derivatives) and an increase in alkyl esters (Figure 5B). Analysis of the relative content of individual compounds showed increases in abundant long-chain aldehydes (C28), alkyl esters (C38, C40, C42), and alcohols (C28), and decreases in almost all salicylates and some unknown compounds. Therefore, overall, the wax composition changed in the transgenic lines, with lipidic components increasing and phenolic components derived from salicylic acid decreasing. This may indicate the impaired function of cutin, as altered partitioning of salicylic acid between the symplast and wax has been found in cutin-defective plants.^42^

### Overexpression of pectate lyase *Ptxt*PL1-27 had little effect on the expression of genes in the cuticle biosynthetic pathway

To determine if changes in the thickness of the cuticle and composition of cutin and wax observed in the transgenic lines were induced by the altered expression of genes responsible for their biosynthesis, we identified eight putative orthologs of *Arabidopsis* genes essential for wax and cutin biosynthesis in the *P. tremula x tremuloides* genome assembly based on phylogenetic analysis (Figure S6) and their high expression in developing leaves (http://plantgenie.org). Comparing their expression in transgenic and WT leaves by RT-qPCR, the only genes that were consistently found to be affected in both transgenic lines were *LACS1*, encoding a long-chain acyl-CoA synthase, which was moderately downregulated, and *ABCG32*, encoding an ABC transporter essential for transport of cuticular precursors and waxes through the plasma membrane, which was slightly upregulated. However, these genes were more affected in line 1051 than 1002. Thus, there was no co-regulation with PEL activity. Other investigated genes, including DC1 encoding putative cutin synthase, DCR-1 and DCR-2 encoding diacylglycerol acyltransferases, and ABCG11 encoding an ABC transporter, were not consistently affected. Therefore, we concluded that the overexpression of *Ptxt*PL1-27 did not induce a downregulation of the entire cutin or wax biosynthetic pathways at the transcript level, although more intricate regulation of specific genes in these pathways cannot be excluded.

### Pectate lyase *Ptxt*PL1-27 increased the lipid content of outer epidermal walls in transgenic plants

**Figure 6:**
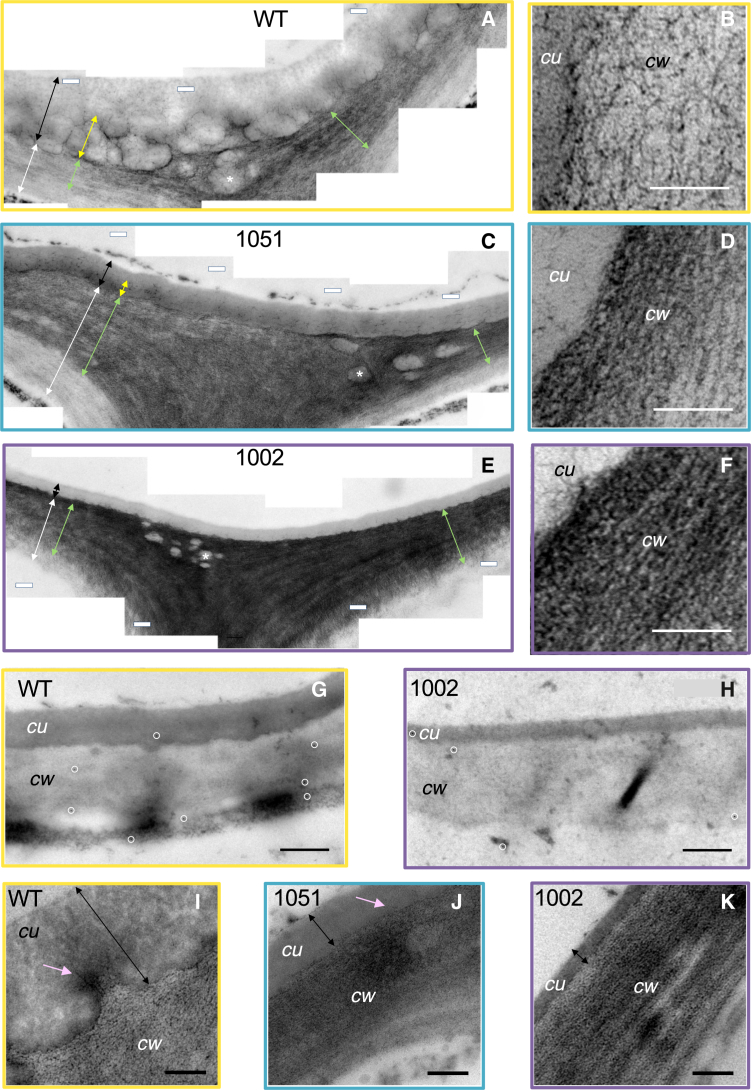
Overexpression of *PtxtPL1-2*7 affects the ultrastructure of the outer cell wall of epidermal cells and the distribution of lipids and polysaccharides (A–F) Representative transmission electron micrographs of sections through the adaxial epidermis treated with uranyl acetate - lead citrate to show lipids in the wild type (WT) (A and B) and overexpressing lines (C–F). (A, C, and E) are composite images of two adjacent epidermal cells; (B, D, and F) are higher magnification images show the boundary between the cuticle (*cu*) and the darkly labeled cell wall (*cw*) layer directly adjacent to the cuticle. White arrows show the primary cell wall, black arrows show the entire cuticle, green arrows show the darkly labeled outer cell wall layer, and yellow arrows in A and C show the inner cuticle layer, which was not discernible in line 1002. Electron translucent inclusions are marked with ∗. (G–K) Representative images show immunolocalization of cutinsomes (signal circled with white ovals) in WT (G) and line 1002 (H), and distribution of polysaccharides detected by PATAg in WT (I) and overexpressing lines (J and K). Pink arrows show polysaccharides in the cuticle layer frequently seen in WT but scarcely in the transgenic lines. Scale bars in A, C, E, I, J, and K = 0.2 μm, in B, D, and F = 0.1 μm and in G and H = 0.5 μm.

After finding no support for the hypothesis that the overexpression of *Ptxt*PL1-27 induces the global downregulation of cutin and wax biosynthetic pathway genes, we tested whether the disruption of HG integrity by PEL could have affected lipid transport through the cell wall. Ultrathin sections through outer epidermal walls prepared for TEM were stained post-embedding with uranyl acetate and lead citrate, which bind free lipids, making them electron-dense. The outer epidermal cell wall in WT plants showed a distinct layer directly adjacent to the cuticle, which was visibly darker than the rest (green arrow in Figures 6A and 6B). Some large electron translucent inclusions in that layer (marked with ∗) were abundant at cell corners and close to the cuticle. As cutin itself exhibited a similar lack of staining by uranyl acetate and lead citrate, these inclusions likely contained polymerized cutin. The presence of the dark-stained outer epidermal cell wall layer indicated lipid accumulation. This layer was broader and darker in the transgenic plants than in WT plants (Figures 6C–6F). On the other hand, the electron translucent inclusions in this layer were much smaller than those in WT. We subsequently used anti-cutinsome antibodies^43^ to detect cutinsome epitopes in the outer cell wall. The signal in WT plants associated with the cell wall-cuticle continuum was very sparse (Figure 6G), in agreement with previous studies using these antibodies,^44^ which precluded the quantitative analysis of gold particles. Therefore, we only analyzed the most highly expressing line, 1002, for qualitative comparison. The anti-cutinsome signal appeared even less abundant in this line than in WT (Figure 6H). We further analyzed the fine distribution of matrix polysaccharides in the cuticle and outer epidermal walls of the transgenic and WT plants using PATAg staining, which revealed dark patches in the inner cuticle and uniform staining of adjacent cell walls in WT (pink arrows, Figure 6I). In comparison, the labeling in the transgenic lines was much darker in the cell walls and scarce or absent in the inner cuticle (Figures 6J and 6K). Moreover, the ultrastructure of the cell wall of transgenic lines was altered compared to WT, with dark PATAg-stained layers that were much better defined. These changes might indicate the accumulation of unpolymerized lipids and compaction of matrix polysaccharides in cell walls adjacent to the cuticle. Overall, these data indicate that the transgenic plants have defects in lipid polymerization and transport through cell walls. The abnormalities are correlated with a low abundance of cutinsome epitopes in the cell wall and cuticle.

## Discussion

Recombinant *Ptxt*PL1-27 exhibits PEL activity on polygalacturonic acid under basic pH (8.5) and in the presence of Ca^2+^ *in vitro.*^34^ When overexpressed *in planta*, it was found to have a clear effect on the cell wall matrix polymer composition of leaves affecting HG content, including acidic and methylesterified HG (Figures 2, 3, and S4; Table 1), whereas compounds with unsaturated -C=C- bonds, which likely correspond to unsaturated HG oligosaccharides generated by PEL (Figure 4), were greatly increased. Additionally, the contents of other polymers, including AGII, RGI, and xyloglucan were affected by PEL, and the extractability of xylan and xyloglucan was increased (Figures 2, 3, and S4; Table 1). Likewise, increased extractability of arabinogalactan, HG, RGI, and xylan was observed in the wood of *Ptxt*PL1-27 overexpressing aspen,^34^ whereas the overexpression of *PtxtPL1-18* in aspen, a PEL from the same clade as *PtxtPL1-27*, resulted in a reduction of acidic HG and RGI epitopes in the primary-walled cambial region tissues.^45^ These findings,^34^^,^^45^ along with data reported here (Figures 2, 3, 4, and S4; Table 1), show that the overexpression of PEL affects HG integrity. They also support the NMR-based evidence that HG is covalently linked to RGI, AGII, and xylan in cell walls^46^ and consequently, these polymers become more extractable from cell walls when HG integrity is affected. The increased xyloglucan extractability in PEL-overexpressing plants suggests the potential involvement of xyloglucan in this network.

The developmental changes observed in PEL-overexpressing plants included decreased internode length and number, resulting in reduced height, accelerated leaf development, increased expansion of epidermal cells, enhanced cell separation and photosynthesis, and altered stomatal function (Figures 1, S1, and S2; Table S1). This highlights the essential role of HG integrity in shoot development, consistent with previous research.^27^^,^^30^^,^^33^^,^^45^^,^^47^^,^^48^^,^^49^^,^^50^^,^^51^^,^^52^ EPG, similarly to PEL, has been shown to inhibit growth and induce cell separation.^28^^,^^29^^,^^32^^,^^52^ However, the phenotypes observed in PEL-overexpressing plants only partially overlap with those of EPG-overexpressing plants. This is not surprising since the two enzymes operate at different pH levels and thus could target different cell wall domains. Moreover, PELs, in contrast to EPGs, do not require water to degrade HG,^26^ suggesting they can potentially function in more hydrophobic environments, such as lignified or cutinized cell walls. Thus, the ectopically expressed PEL could target HG integrity in lignified and cutinized cell walls more efficiently than EPG. This study highlights the significant impact of PEL overexpression on leaf epidermal cells that form the cuticle.

We found that the ectopic overexpression of PEL only slightly affected cuticle permeability but drastically altered cuticle development in aspen. The highly expressing line (1002) displayed precocious cork deposition (Figure 1D), reminiscent of the phenotype observed in tomato fruits with impaired cutin polymerization.^53^ Cuticle permeability (but not thickness) was affected in *Arabidopsis* and apple lines overexpressing fungal EPG, and the primary mechanism underlying the cuticle permeability defect appeared to be related to reduced cellular adhesion, dependent on the functions of *At*PRX71 and *At*ESMERALDA1 and stress responses.^31^^,^^32^ Whereas it is likely that the ectopic PEL overexpression also induced stress responses, its effects on cuticle were clearly different than those of EPG, as it reduced cuticle thickness, altered cuticle morphology, and cutin and wax compositions, whereas cuticle permeability was only slightly affected (Figures 4, 5, 6, S3, and S4). The reduced cuticle thickness could not be explained by the transcriptomic suppression of the cutin or wax biosynthetic machinery (Figure S6) because only one gene among eight key genes investigated here, *PtxtLACS1*, potentially involved in 16C and very long-chain FA biosynthesis,^54^ showed slight downregulation in the transgenic lines (by 20% of the WT level in the most affected line). Such low-level downregulation is unlikely to cause the major reduction in cuticle thickness observed in PEL-overexpressing plants. It is, however, possible that the expression of *PtxtLACS1* or that of other genes in this pathway, which we did not analyze here, is regulated in response to cuticle defects, cell wall defects, or some other effects of PEL. Considering the pleiotropic effects of ectopic PEL overexpression, many indirect effects are likely to co-occur.

It is also possible that PEL has a more direct effect on cuticle development. As our observations revealed significant alterations in the outer epidermal cell wall ultrastructure, changes in the distribution of de-esterified HG, xyloglucan, and PATAg-reactive polysaccharides, and a dramatic increase in the content of lead citrate and uranyl acetate-reactive compounds, likely lipids (Figures 3, 4, 5, and 6), we propose that a defective transport of cutin monomers and oligomers through the cell wall, resulting in their accumulation in the cell wall, could be mainly responsible for the cuticle defects in PEL-overexpressing plants. According to this hypothesis, PEL, by disrupting HG, could interfere with the cutinsome outer shell,^11^ thereby affecting the transport and assembly of cutin precursors in cutinsomes through the cell wall. This hypothesis is further supported by the altered distribution of acidic HG in the cell wall directly adjacent to the cuticle (Figure 3) and by the reduced presence of epitopes recognized by anti-cutinsome antibodies^43^ in the cell wall-cuticle continuum of the transgenic line 1002 compared to WT (Figures 6G and 6H). Considering all the above data, we propose that the integrity of acidic HG is essential for cutin monomer transport through the cell wall, cutin polymerization, and cuticle assembly.

### Limitations of the study

Ectopic expression of *Ptxt*PL1-27 produces pleiotropic effects; therefore, our interpretation of the mechanism of the observed responses linking cuticle defects with homogalacturonan integrity and cutinsome-mediated lipid transport should be taken with caution. Although we did not find support for the idea that *Ptxt*PL1-27 indirectly induces changes in gene expression leading to abnormal cuticle biosynthesis, this possibility cannot be entirely excluded because our analysis of gene expression was done in whole leaves, whereas the genes could be affected specifically in the epidermis. Therefore, tissue-specific gene expression analysis would be helpful to confirm these results at higher resolution. Despite these limitations, the results consistently support the central conclusion that *Ptxt*PL1-27 alters cuticle structure and function in developing leaves.

## Resource availability

### Lead contact

Further information and requests for resources and reagents should be directed to and will be fulfilled by the Lead Contact, Ewa Mellerowicz (ewa.mellerowicz@slu.se).

### Materials availability

This study did not generate new reagents.

### Data and code availability

• All data generated for this article are available as main and supplemental information.
• This study did not generate any new code.
• Any additional information required to reanalyze the data reported in this article is available from the lead contact upon request.

## Acknowledgments

We thank Prof. Antonio Heredia for the gift of anti-cutinsome antibodies, Sushree Sangita Mohanty for help with cell wall analyses, Dr. Prashant Pawar and Dr. Christine Ratke for wax collection. We acknowledge the participation of the following analytical platforms: CCRC Analytical Services for linkage analyses, the Swedish Metabolomics Center in Umeå for untargeted metabolomics, the Vibrational Spectroscopy Core Facility for FTIR spectroscopy, the UPSC transgenic facility for the transformation and propagation of the transgenic lines, Dr. Agnieszka Ziółkowska and the Umeå Center for Electron Microscopy for electron microscopy analyses, and the Umeå Plant Science Center Microscopy Facility for light microscopy analysis. A.K.B. acknowledges support from the 10.13039/100014456Center for Bioenergy Innovation (CBI), U.S. Department of Energy, Office of Science, Biological and Environmental Research Program, under Award Number ERKP886. H.V.S. acknowledges support from the Joint BioEnergy Institute (JBEI), U.S. Department of Energy, Office of Science, Biological and Environmental Research Program, through contract DE-AC02-05CH11231 between 10.13039/100006235Lawrence Berkeley National Laboratory and the U.S. Department of Energy. P.S. acknowledges financial support from 10.13039/100004410European Molecular Biology Organization, award number EMBO-STF-7036. This study was supported by funding from 10.13039/501100001858VINNOVA, 10.13039/501100004063Knut and Alice Wallenberg Foundation, SSF, VR, and Formas to E.J.M.

## Author contributions

Morphology of the transgenic lines (A.K.B., M.M., A.B., and P.I.), RNA and activity assays (A.K.B.), cell wall sugar composition (A.K.B., J.H., and H.V.S.), glycome profiling (S.P. and M.H.), linkage analysis (A.K.B.), FTIR spectroscopy and data analysis (P.I. and A.G.), immunolocalization (A.B., M.M., P.S., and M.D.-M.), ultrastructure (P.S. and M.D.-M.), cuticle analysis (I.A., S.K., T.M., and M.M.), wax analysis and integration of the results with gene expression (J.-P.F.-M. and A.A.), gene identification and RT-PCR (A.K.B. and V.K.), conceptualization, project coordination, first draft (A.K.B. and E.J.M.) and final article compilation (E.J.M. with contributions from all authors).

## Declaration of interests

Several authors have new current affiliations not listed here, which started after the data for this article were generated. These affiliations do not have any relationship with the work presented here.

## STAR★Methods

### Key resources table

**Table:** 

REAGENT or RESOURCE	SOURCE	IDENTIFIER
**Antibodies**		

Plant cell wall glycan-directed mAbs	http://www.carbosource.net; http://glycomics.ccrc.uga.edu/wall2/jsp/abIndex.jsp	Pattathil et al.,^36^ Thorne et al.^55^
anti-cutinosomes	Prof. Antonio Heredia	Domínguez et al.^43^
Secondary antibody: Anti-Mouse or Anti-Rat	Sigma Aldrich	Cat#A4416 or Cat#A9037
Mouse monoclonal CCRC M1	CarboSource	RRID: AB_CCRCM1
Rat monoclonal JIM5	PlantProbes	Cat#JIM5
EM Goat anti-Rat IgG (H&L): 10 nm Gold	BBInternational	EM.GAT
Goat anti-Mouse IgG: 2 and 10 nm Gold	BBInternational	RRID: AB_EM Goat anti-Mouse IgG: 10 nm Gold

**Chemicals, peptides, and recombinant proteins**		

Chloroform	ACS, ISO, Reag. Ph Eur Chloroform EMSURE	CAS 67-66-3
n-tetracosane (C24)	Sigma Aldrich	CAS 646-31-1
N,O-bis(trimethylsilyl)trifluoroactamide (BSTFA)	Sigma Aldrich	CAS 25561-30-2
Pyridine	ACS Reag., Sigma Aldrich	CAS 110-86-1
TMB Peroxidase Substrate	Vector Laboratories, Burlingame, CA	Cat#Kit SK-4400
Dialysis membranes: Spectra/Por® 3, MWCO 3500	Spectrum Laboratories	Cat#28170-166
1 M methanolic-HCl	Sigma Aldrich	Cat#90964
Zinc Chloride	Fisher Scientific	CAS number: **7646-85-7**
Hydrochloric acid	Sigma Aldrich	CAS number: **7647-01-0**
Phosphorus pentoxide	Sigma Aldrich	CAS number: **1314-56-3**
Sodium methoxide solution, 0.5 M CH3ONa in methanol (0.5N)	Sigma-Aldrich Sweden AB	CAS number: **124-41-4**
Methanol	Sigma-Aldrich	CAS number: **67-56-1**
LRW resin, hard grade - catalyzed	TAAB essentials for microscopy, England, UK	L009

**Experimental models: Organisms/strains**		

*Populus tremula x tremuloides*	Umeå Plant Science Center, Umeå, Sweden	Clone T89

**Software and algorithms**		

cheminfo	http://www.cheminfo.org/Chemistry/Database/PubChem/Search_by_exact_mass_in_PubChem/index.html	N/A
lipidmaps	https://www.lipidmaps.org/	N/A
SIMCA-P+	version 12, Umetrics AB, Umeå, Sweden	N/A
OPUS	version 6.5, Bruker Optik GmbH, Ettlingen, Germany	N/A
The “R” Program for Statistical Computing	https://www.r-project.org/	N/A
MorphoGraphX	https://morphographx.org/	Strauss et al.^56^

### Experimental model and study participant details

#### Plant lines, growth conditions and sampling

Transgenic hybrid aspen (*Populus tremula* L. x *tremuloides* Michx.) plants clone T89 overexpressing *PtxtPL1-27* were described previously.^34^ They carried the coding sequence of *PtxtPL1-27* fused with the *35S CaMV* promoter. Transgenic lines 808, 302, 1051 and 1002 and WT were *in vitro* propagated and clonal replicates were grown in a glasshouse under an 18/6 h photoperiod, 60% humidity and 20°C, with supplementary lighting (Osram Powerstar HQI-BT 400 W/D, Osram, Germany) switched on when the natural light fell below 20 Wm^-2^ during the photoperiod. Plants were watered daily and fertilized with a complete nutrient solution (SuperbaS, Supra HydrO AB, Landskrona, Sweden) once a week.

For different analyses, the leaves were sampled at defined developmental stages: i) expanding leaves (typically leaves 6–9); ii) young expanded leaves (typically leaf 10–15); and iii) mature leaves (typically leaf 16 and older).

### Method details

#### Stomatal conductance and transpiration

Stomatal conductance and transpiration rates were measured using a portable infrared gas analyzer LI-COR 6400XT (LI-COR Biosciences UK Ltd, www.licor.com) in mature leaves.

#### Transgene expression analyses

Apical buds including internodes 1–10 with developing leaves were flash frozen in liquid N_2_ and ground to a fine powder using a mortar and pestle. Total RNA and different protein fractions were extracted and used for transgene expression and enzymatic activity analyses, respectively, as previously described.^34^

#### Cell wall analyses

Mature leaves had their main veins removed and were ground in a mortar with liquid nitrogen. The obtained material was suspended in 70% ethanol, and after centrifugation, the pellet was resuspended in methanol:chloroform 1:1 (v/v), centrifuged, washed three times with acetone with intervening centrifugation and dried overnight under vacuum. The resulting alcohol-insoluble residue (AIR) (10 mg) was subjected to sequential extraction using 1 mL of each solvent, as previously described.^36^ The AIR was destarched by treating with *Bacillus* α-amylase (type II-A from *Bacillus*, Sigma-Aldrich A6380, St Louis, MO, USA) at 5000 units per g of AIR for 16 h at 37°C with constant shaking to obtain the amylase fraction. Subsequently, the residue was washed with deionized water and treated with EPG I and II from *Aspergillus niger*^57^ and PME from *A. niger*^58^ at ∼1.0 unit per 100 mg of AIR in 50 mM sodium acetate at pH 5.0 for 24 h at room temperature to obtain the EPG/PME fraction. The pellet was sequentially suspended in 50 mM Na_2_CO_3_ containing 0.5% (w/v) NaBH_4_, (pH 10.0), 1 M KOH with 1% (w/v) NaBH_4_ and 4 M KOH with 1% (w/v) NaBH_4_ for 24 h each with constant shaking at 100 rpm and centrifugation at 4000*g* for 15 min at room temperature. The resulting supernatants were collected as the sodium carbonate extract, 1 M KOH extract and 4 M KOH extract, respectively. All the extracts were prepared in duplicates from AIR obtained from two pools of 5 trees per each line. They were dialyzed against six changes of de-ionized water at room temperature for 72 h.

For the sugar composition analysis, the AIR (∼2 mg) and fractionated lyophilized polysaccharides (300 μg) were subjected to methanolysis-trimethylsilyl (TMS) analysis.^59^^,^^60^ They were hydrolyzed for 18 h at 80°C in 1 M methanolic-HCl, cooled to room temperature, dried with anhydrous methanol and derivatized with 200 μL of TriSil Reagent (Pierce-Endogen, Rockford, IL, USA) for 20 min at 80°C. Afterward, the samples were evaporated under a stream of dry air, suspended in 2 mL hexane and 1 μL of sample was injected for GC-MS into an Agilent 7890A gas chromatograph interfaced to a 5975C mass selective detector.^60^ The analysis was conducted on two biological and two technical replicates.

For the glycosyl linkage analysis, about 1.2 mg of the fractionated lyophilized sample was suspended in 200 μL of dimethyl sulfoxide and then stirred for two days at 25°C. The samples were permethylated using potassium dimsyl anion and iodomethane, and the permethylated uronic acids were reduced using lithium borodeuteride.^61^ An additional permethylation was carried out by two rounds of treatment with sodium hydroxide for 15 min and methyl iodide in dry dimethyl sulfoxide for 45 min.^62^ The permethylated material was hydrolyzed using 2 M tri-fluoroacetic acid (TFA) for 2 h in sealed tubes at 121°C, reduced with NaBD_4_ and acetylated using acetic anhydride/TFA.^59^^,^^61^ The resulting partially methylated alditol acetates were analyzed by GC-MS^63^ using an Agilent 7890A gas chromatograph interfaced to a 5975C MSD and operated in the electron impact ionization mode, and separation was performed on a 30 m Supelco SP-2331 bonded phase fused silica capillary column.

Lyophilized sequential cell wall extracts (prepared in duplicates) were subjected to enzyme-linked immunosorbent assay (ELISA-based mAb screenings), as previously described.^36^^,^^61^ Plant cell wall glycan-directed mAbs were obtained from laboratory stocks (CCRC, JIM and MAC series) maintained by the Complex Carbohydrate Research Center (CarboSource Services; http://www.carbosource.net). Supplemental information on the mAbs used in this study was described earlier.^36^^,^^55^

#### Light microscopy

To analyze stomatal function, epidermal peels from young expanded leaves were incubated in 25 mM MES-KOH and 10 mM KCl at pH 7.0 in the light for 2 h to induce stomatal opening, then placed in 5 mM CaCl_2_ for 2 h to induce stomatal closure. The peels were mounted in their medium and photographed immediately. The experiment was repeated using different trees with similar results.

For analysis of the cuticle morphology without fixation, fresh sections of young expanded leaf (leaf 10) in the region of the midrib were de-aerated in water for 1 h, stained with 0.01% ruthenium red for 10 min and then washed and mounted in water.

For analysis of the adaxial and abaxial epidermis during leaf development, basal pieces of expanding and young expanded leaves (leaves 6 and 10, respectively) close to the midrib were boiled in 95% ethanol to remove chlorophyll, washed three times with 95% ethanol, stained with 0.1% toluidine blue for 0.5 h, washed again in 95% ethanol and mounted in glycerol.

For analysis of the leaf anatomy, basal pieces of young expanded leaf 10 were fixed in FAA (5% formaldehyde, 5% acetic acid and 50% ethanol), rinsed in 50% ethanol and embedded in paraffin wax.^64^ Transverse 10 μm thick sections were obtained using a rotary microtome (RM2135, Leica, Wetzlar, Germany), de-waxed, stained with Alcian Blue-Safranin O (Sigma-Aldrich)^65^ and mounted in glycerol.

Sections were analyzed using an Axioplan 2 microscope equipped with an AxioVision camera (Zeiss, Oberkochen, Germany) or a BX-50 (Olympus Optical Co.) microscope connected to a DP71 digital camera (Olympus Optical Co., Tokyo, Japan).

Image processing and cell segmentation were performed as previously described^66^ using MorphoGraphX.^56^ Images were pre-processed by adjusting saturation, contrast, and sharpness before segmentation. A surface mesh was generated using the Marching Cubes Surface algorithm, and cell segmentation was performed with the Watershed Segmentation process. The Smooth Mesh process was applied to refine cell contours. Cell area was analyzed using MorphoGraphX tools (https://morphographx.org/). The data are based on three biological replicates (trees) per condition, each tree represented by up to 9 images, and approximately 9 cells were measured on each image.

#### Transmission electron microscopy and immunolocalization

Small leaf fragments, with areas of approximately 1 mm^2^, from young expanded leaves (leaf 10) were fixed in an ice-cold solution containing 4% paraformaldehyde, 0.05% glutaraldehyde (GA) and 100 mM phosphate buffer at pH 7.2, vacuum-treated in a desiccator for 1 h, transferred to 4°C and left overnight, rinsed in phosphate buffer and dehydrated using an ethanol series ranging from 10% to 99.5% (v/v). The ethanol was gradually replaced with LR White resin (hard grade, catalyzed; TAAB Laboratories Equipment Ltd., Berkshire, UK), and the samples were polymerized in polypropylene capsules (TAAB) at 60°C for 12 h. Ultrathin sections (70 nm thick) were prepared using a Reichart Ultracut microtome and mounted on formvar-coated copper grids (Sigma-Aldrich).^67^

For lipid staining, sections were post-embedding stained with 5% uranyl acetate (EMS, Hatfield, PA 19440) in water for 1 h, and lead citrate (TAAB Laboratories, Aldermaston, UK) for 6 min.^68^

For immunolocalization experiments, sections were first blocked in a 3% bovine serum albumin (BSA) solution in phosphate-buffered saline (Sigma-Aldrich), incubated with primary antibodies for 45 min (see Table S7 for details regarding antibodies), rinsed in 1% BSA and 0.05% Tween in PBS and incubated with secondary antibodies overnight. After incubation, the grids were rinsed in water and allowed to dry. For contrast enhancement, the grids were treated with 5% uranyl acetate (Sigma-Aldrich), washed under running water and dried.^67^

For analysis of cell wall polysaccharides, ultrathin sections were placed on gold grids and subjected to periodic acid-thiocarbohydrazide-silver proteinate (PATAg) staining.^69^ Briefly, the grids were treated for 30 min with 1% periodic acid, then rinsed three times in distilled water and incubated in 20% (v/v) acetic acid containing 0.2% thiocarbohydrazide for 48 h, followed by incubation in a series of decreasing concentrations of acetic acid and three subsequent washes with water. Finally, the grids were floated on 1% silver proteinate (Fluka Biochemica, Buchs, Switzerland) for 30 min in the dark and then rinsed three times with water.

The sections of two trees per line were examined with a Jeol 1230 transmission electron microscope (JEOL Ltd, Tokyo, Japan) or a TALOS L120C transmission electron microscope (Thermo Fisher Scientific, Gothenburg, Sweden), operated at 80 kV.

#### Attenuated total reflectance Fourier transform infrared spectroscopic analysis of leaf surface

Mature leaves (leaf 16) were harvested from 4 to 6 trees per line, wrapped in aluminum foil and stored between two layers of dry ice before storage at −20°C. Attenuated total reflectance Fourier transform infrared (ATR FTIR) spectroscopic analysis was performed using a Bruker Equinox 55 system (Bruker Optik GmbH, Ettlingen, Germany) with a room temperature deuterated triglycine sulfate detector. Each leaf was firmly pressed with the upper epidermis against a diamond ATR crystal (SensIR Technologies). 512 scans were conducted, covering the spectral range 5200 - 400 cm ^−1^ with 4 cm^−1^ spectral resolution and a zero-filling factor of two. A spectrum of the empty ATR accessory without leaf was used as the background, which was recorded with the same parameters as the leaf spectra but using 256 scans. On each leaf, spectra were recorded at three different positions using the OPUS software package (version 6.5, Bruker Optik GmbH, Ettlingen, Germany), and the data were exported as ASCII text files. The individual spectra were compiled into a single file and processed using a free, open-source, MATLAB based graphical user interface^70^ available at the Vibrational Spectroscopy Core Facility at Umeå University. Processing included cutting the spectral region to 890–4000 cm^−1^, followed by asymmetrical least squares baseline correction (lambda = 10,000; *p* = 0.001) and total area normalization over the cut spectral range. Multivariate data analyses were performed using SIMCA-P+ (version 12, Umetrics AB, Umeå, Sweden), relying on built-in functions. Initial principal component analysis (PCA) with autofit was followed by pairwise orthogonal projections to latent structures – discriminant analysis (OPLS-DA). The number of orthogonal components in OPLS-DA was manually set to one to avoid overfitting and facilitate direct comparison between models.

#### Cutin analysis

The cuticle of mature leaves was isolated from small pieces (1.5–2 cm^2^) of leaf blade treated with chloroform to remove waxes, followed by incubation with ZnCl_2_-HCl reagent (30 g ZnCl_2_ in 51 mL conc. HCl) with gentle agitation for 24–36 h at room temperature.^71^ Detached fragments of cuticle were washed with distilled water, air dried and then vacuum dried over P_2_O_5_ desiccant overnight. The samples were subsequently methanolized with freshly prepared ice-cold 50 mM NaOCH_3_ in dry methanol, flushed with a stream of nitrogen for 10–15 s, immediately sealed in tubes fitted with Teflon coated caps, incubated for 24 h at 85°C and vortexed. Afterward, the debris was filtered out using 0.45 μm PTFE filters,^72^ followed by two washes with methanol. The combined filtrates were evaporated at 40°C under a stream of nitrogen to dryness, and 10 mg of this material was used for lipid extraction in 1000 μL of MeOH:CHCl_3_ (1:1, v/v), as previously described.^73^ The chloroform phase (2 μL) was analyzed by an untargeted lipidomics approach using a liquid chromatography quadrupole time-of-flight mass spectrometry instrument (Agilent, Santa Clara, CA) operated in the negative mode with a mass range of 100–1700 *m*/*z*, as previously described.^74^ Auto MS/MS analysis was performed under the same chromatographic conditions to identify possible C16 or C18 monomer fragments (containing OH or epoxy groups) produced by methanolysis of cutin ester groups, using collision energies of 10, 20 and 40 V. Putatively annotated compounds are listed in Table S4. For metabolites showing a *p*-value ≤0.05 and fold change ≥2 (Table S5), cheminfo (http://www.cheminfo.org/Chemistry/Database/PubChem/Search_by_exact_mass_in_PubChem/index.html) and lipidmaps (https://www.lipidmaps.org/) databases were used for annotation and grouping into different classes of lipids.

#### Cuticular wax extraction and GC-MS analysis

Cuticular wax analysis was performed as previously described using mature leaves.^41^ Leaf discs were extracted in chloroform for 30 s with gentle shaking and *n*-tetracosane (0.2 mg/mL) was added to the extracts as an internal standard. Samples were derivatized with N,O-bis(trimethylsilyl)trifluoroacetamide (BSTFA) and pyridine (1:1 v/v) at 70°C for 40 min. The qualitative and quantitative composition of the cuticular waxes was analyzed with a GC system (Trace GC, Thermo Fisher Scientific) connected to an electron impact MS detector (DSQ2; Thermo Fisher Scientific) set at 70 eV and *m*/*z* 40–850 Da (±0.5 Da). Samples were injected using a solvent split mode (50 mL/min of split flow) in a programmable temperature vaporization injector, and compounds were separated in a Zebron DB-1 column (Phenomenex ZB-1MS, 30 m length, 0.25 mm I.D. and 0.50 μm film thickness). GC was carried out in an oven programmed as follows: 0.5 min at 100°C, 30°C min^−1^ to 210°C, 0 min at 210°C, 5°C min^−1^ to 330°C and 35 min at 330°C, with helium flow at 1.2 mL/min. Kevat’s indices were used to identify wax constituents in the total ion chromatogram. Mass spectral comparison between wax constituents and both authentic standards and data from the literature were used for identification. Detailed information about the specific fragments for each wax compound is available in the literature.^41^ The percentage of total signal for each wax compound and wax group was calculated considering the total signal as the total area of both known and unknown peak compounds. The data were centered $\left(\tilde{x}\text{ij}=\text{xij}-\bar{x}i\right)$ and scaled using range scaling $\left(\tilde{x}\text{ij}=\frac{\text{xij}-\bar{x}i}{\mathrm{Max}\left(\text{xj}\right)-\mathrm{Min}\left(\text{xj}\right)}\right)$ prior to statistical analysis.^75^

#### Quantitative PCR analysis of cuticle biosynthetic genes

*P. tremula x tremuloides* clone T89 gene models corresponding to known cutin biosynthetic genes in *Arabidopsis* and tomato were identified by phylogenetic analysis using Phylogeny.fr (http://www.phylogeny.fr/simple_phylogeny.cgi), and their expression in developing leaves was verified using an RNA-seq expression dataset for *P. tremula* at Plantgenie (https://plantgenie.org/). Total RNA was extracted from frozen ground mature leaves of three trees from each line using the RNeasy PlantMiniKit (Qiagen, Kista, Sweden), DNase treatment was performed using the DNAfree kit (Invitrogen, Thermo Fisher Scientific) and cDNA was synthesized using the iScript cDNA synthesis kit (Bio-Rad, Solna, Sweden), following the manufacturers’ protocols. Quantitative PCR (qPCR) was performed using the Light Cycler 480 SYBR Green I master mix (Roche AB, Solna, Sweden) in a Light Cycler 480 II instrument (Roche) as per the manufacturer’s instructions. The relative change in expression levels of selected genes in the transgenic lines compared to WT was calculated^76^ using a ubiquitin-related gene as a reference gene. Primers and their efficiencies are listed in Table S8.

### Quantification and statistical analyses

Details of statistical analyses are listed in figure legends.

## Supplemental information

Document S1. Figures S1–S6 and Tables S1, S3, S6, and S7

Table S2. Linkage analysis in different cell wall fractions prepared from leaves of wild-type plants (WT) and transgenic lines overexpressing PtxtPL1-27

Table S4. Metabolite profiling of cutin monomers by liquid chromatography quadrupole time-of-flight mass spectrometry (LC-QTOF-MS)

Table S5. Aliphatic compounds in the depolymerized cuticle that were differentially abundant in the transgenic lines and wild type

Table S8. Primers used for RT-PCR of cutin biosynthetic genes
