# Supplementary material for: Ectopic expression of pectate lyase PtxtPL1-27 in aspen affects leaf cuticle development
Source: iScience. 2025 Nov 6;28(12):113963. doi: 10.1016/j.isci.2025.113963 (PMC12671348; doi:10.1016/j.isci.2025.113963)
Supplement: Document S1. Figures S1–S6 and Tables S1, S3, S6, and S7 [file mmc1.pdf]

iScience, Volume *28*

Supplemental information

Ectopic expression of pectate lyase

*Ptxt*PL1-27 in aspen affects

leaf cuticle development

Ajaya K. Biswal, Alicja Banasiak, Josefina-Patricia Fernández-Moreno, Madhusree Mitra, Jesper Harholt, Marta Derba-Maceluch, Mateusz Majda, Sunita Kushwah, Vikash Kumar, Ilka Abreu, Pramod Sivan, Sivakumar Pattathil, Peter Immerzeel, András Gorzsás, Thomas Moritz, Michael G. Hahn, Henrik Vibe Scheller, Asaph Aharoni, and Ewa J. Mellerowicz

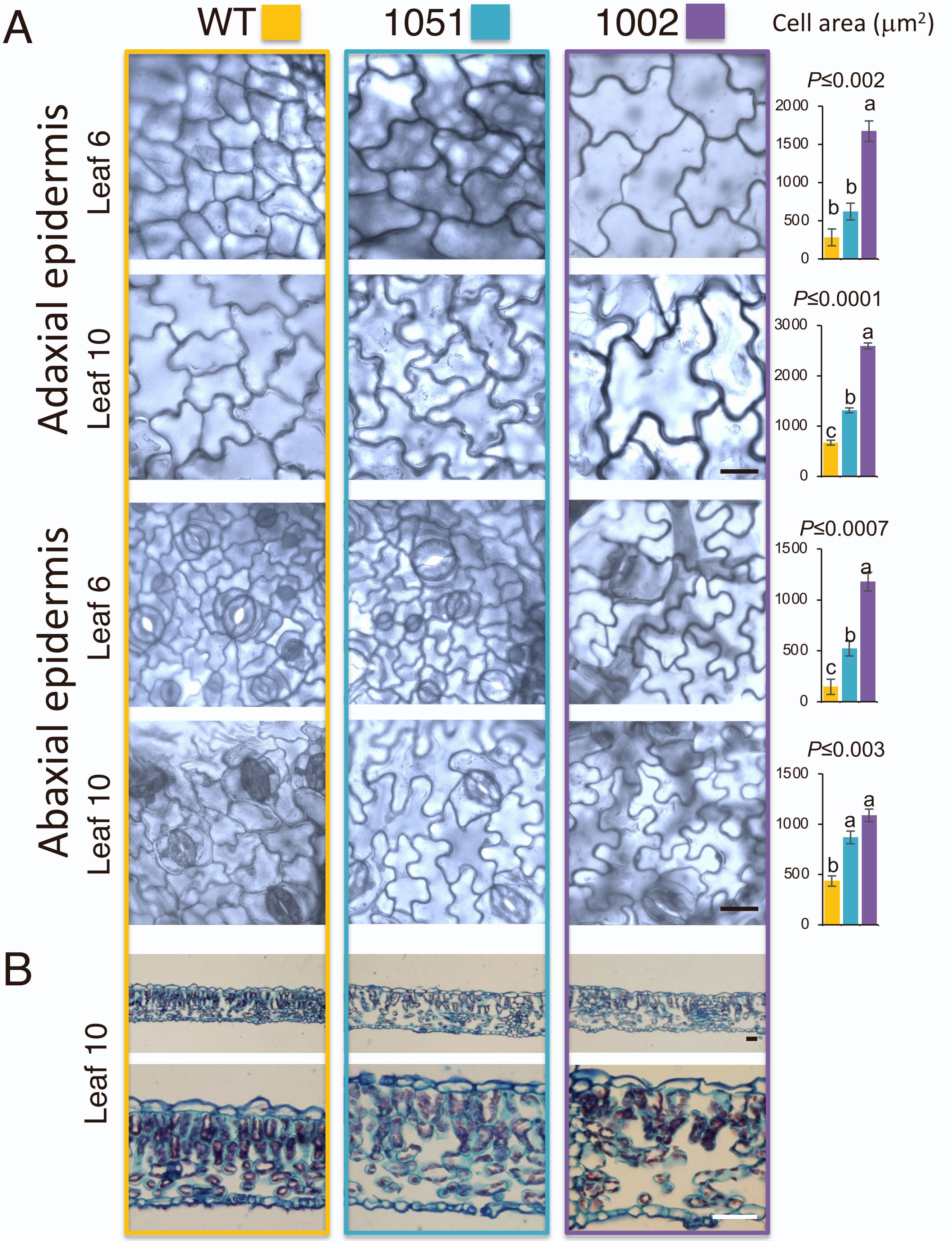

Figure S1. **Overexpression of *PtxtPL1-27* alters leaf development in hybrid aspen. (A)** Images of adaxial and abaxial epidermis in expanding (6) and young expanded (10) leaves of transgenic lines 1002 and 1051 and in wild-type (WT), and the corresponding epidermal cell areas. Scale bars = 20 μm. Bar graphs show means ± SE, n=3 trees. Means accompanied by the different letters are significantly different ($P \leq 0.05$, Tukey test). P values above the bars show the significance of the difference between the transgenic and WT plants (contrast analysis). **(B)** Sections of leaf 10 stained with alcian blue-safranin showing reduced cell adhesion in transgenic lines compared to WT. Upper and lower rows correspond to low and high magnification, respectively. Scale bars = 50 μm.

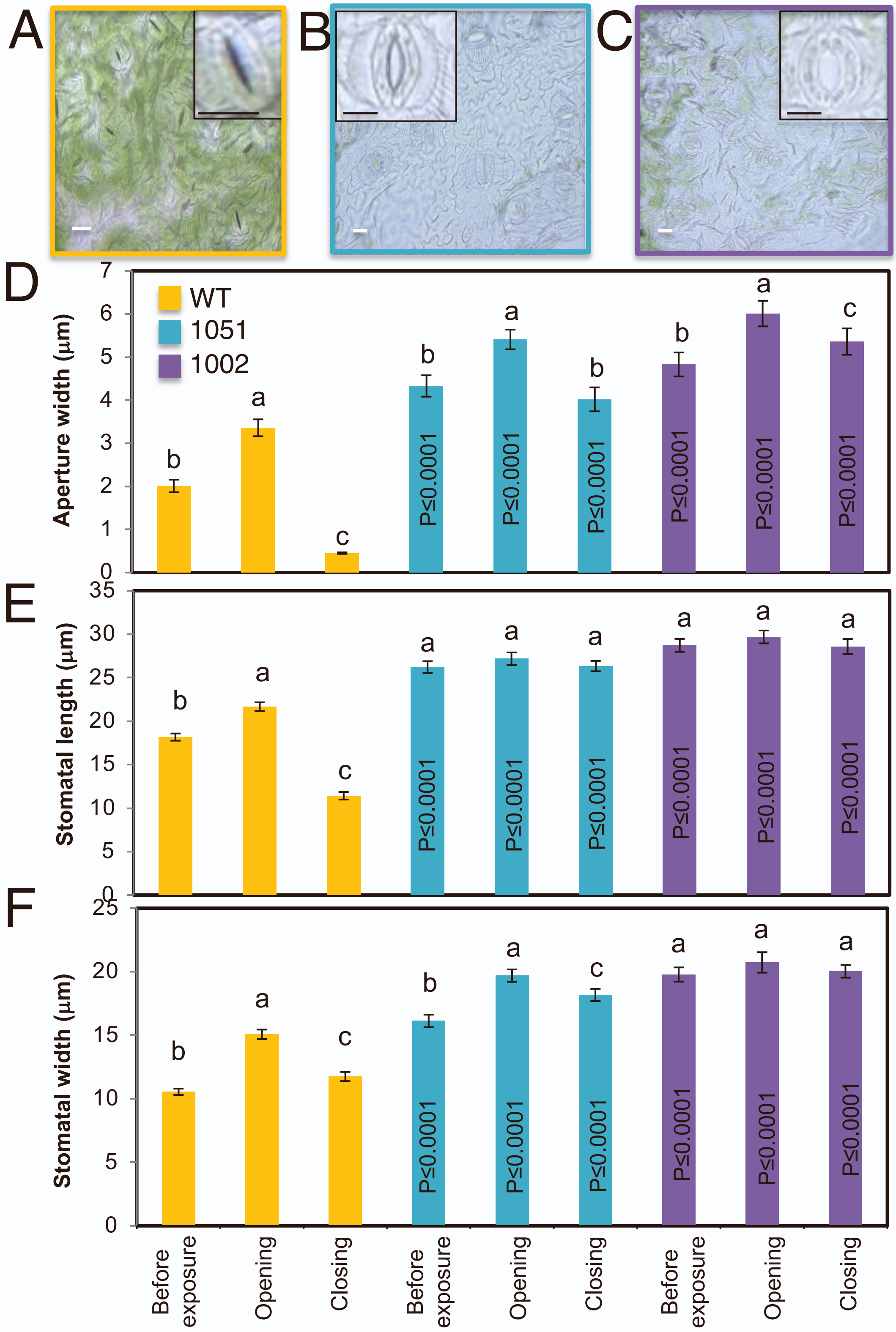

Figure S2. Overexpression of *PtxtP*L1-27 increases stomatal sizes and affects stomatal function. (A-F) The effect of *PtxtP*L1-27 on stomatal sizes and calcium induced stomatal closure in young expanded leaves. After incubation in 25 mM MES-KOH, 10 mM KCl, pH 7.0, the leaf epidermal peels were exposed to light for 2 h to induce stomatal opening or placed in 5 mM $CaCl_2$ for 2 h to induce stomatal closure. Images of stomata after $CaCl_2$ exposure in wild-type (WT) **(A)**, and transgenic lines 1051 **(B)** and 1002 **(C)**. Stomatal aperature **(D)**, length **(E)** & width **(F)** in WT and *PtxtPL1-27* overexpressing lines atfer stomatal opening- and closing-inducing treatments. Mean ± SE, N=80; different letters indicate significant treatment effect within each line (Tukey test, P≤0.05), P values show significance of the difference between each line and the WT (Dunett test). Scale bars = 10 µm (A,B,C).

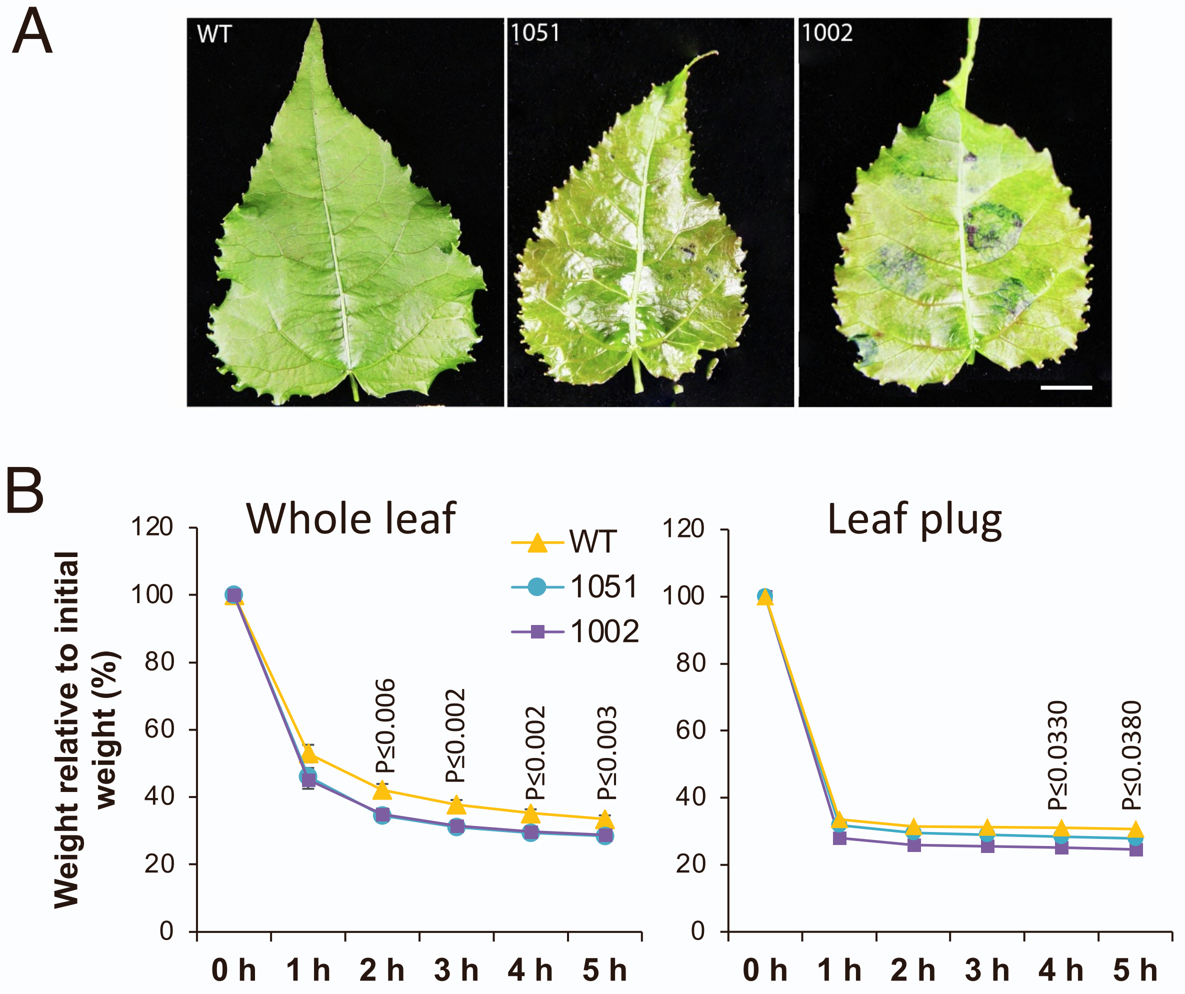

Figure S3. Overexpression of *PtxtP*L1-27 affects cuticle permeability and leaf water loss. (A) Transgenic lines have compromised cuticle permeability as shown by toluidine blue staining in expanding leaves. **(B)** Kinetics of leaf water loss. Detached young expanded leaves or leaf plugs were left in the ambient atmosphere (22 ºC and RH approx. 50%) and their fresh weight was recorded for 5 h. Mean ± SE, N=5. P values correspond to significance of the difference between both transgenic lines and WT (post-ANOVA contrast). Scale bar = 2 cm.

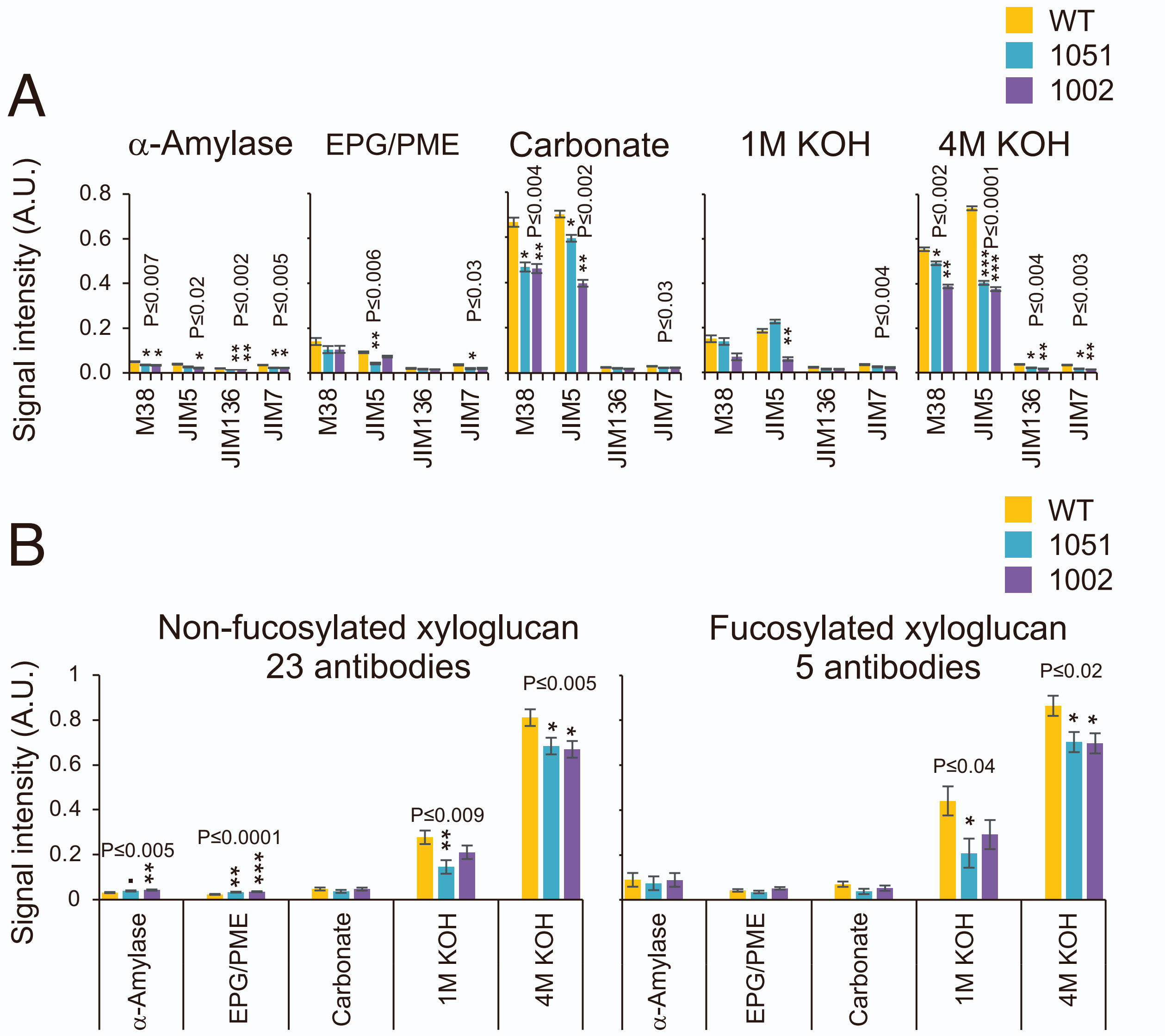

Figure S4. Statistical analysis of signals from the glycome analysis homogalacturonan (HG)- and xyloglucan-specific monoclonal antibodies. (A) Signals from individual antibodies specific for different epitopes of HG backbone. **(B)** Average signals from indicated number of individual antibodies specific for non-fucosylated and fucosylated xyloglucan. Mean ± SD, N=2 for **A**, corresponding to two biological replicates, or 23 – 5 for **B**, corresponding to the number of different antibodies used, as indicated. P values correspond to significance of the difference between both transgenic lines and WT (post-ANOVA contrast). Asterisks indicate means significantly different from WT according to the Dunnett's test (▪ - P ≤ 0.1, * - P ≤ 0.05, ** - P ≤ 0.01, *** - P ≤ 0.0=1). P values above the bars correspond to the post-ANOVA contrast analysis comparing both transgenic lines to wild type (WT). A.U. – arbitrary units. Related to Figure 2B.

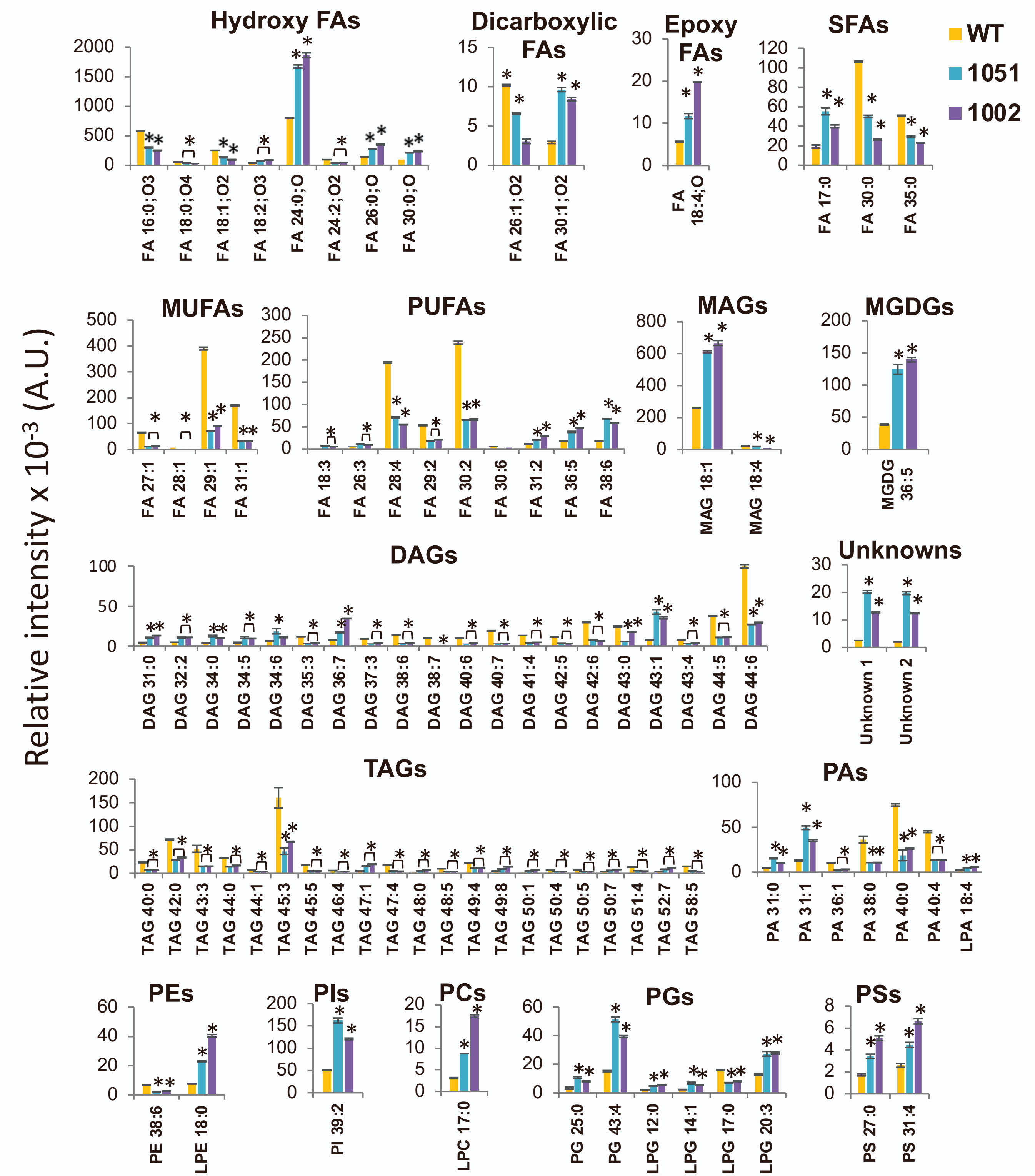

Figure S5. Relative concentrations of the different lipids identified in depolymerized cuticles is significantly affected by *Ptxt*PL1-27 expression. All detected compounds are listed in LC-MS datasets in Supplemental Table S6. Mean ± SE, N=3 biological replicates. Asterisks indicate means significantly different from WT (fold change ≥ 2, P ≤ 0.05, Dunnett's test for individual lines, t-test for combined transgenic lines versus WT – indicated by a clump). DAGs: Diacylglycerols; FAs: Fatty acids; FOHs: Fatty alcohols; LPAs: Lysophosphatidic acids; LPCs: Lysophosphatidylcholines; LPEs: Lysophosphatidylethanolamines; LPGs: Lysophosphatidylglycerols; MAGs: Monoacylglycerols; MGDGs: Monogalactosyldiacylglycerols; MUFAs: Monounsaturated fatty acids; PAs: Phosphatidic acids; PEs: Phosphatidylethanolamines; PGs: Phosphatidylglycerols; PIs: Phosphatidylinositols; PSs: Phosphatidylserines; PUFAs: Polyunsaturated fatty acids; SFAs: Saturated fatty acids; TAGs: Triacylglycerols;

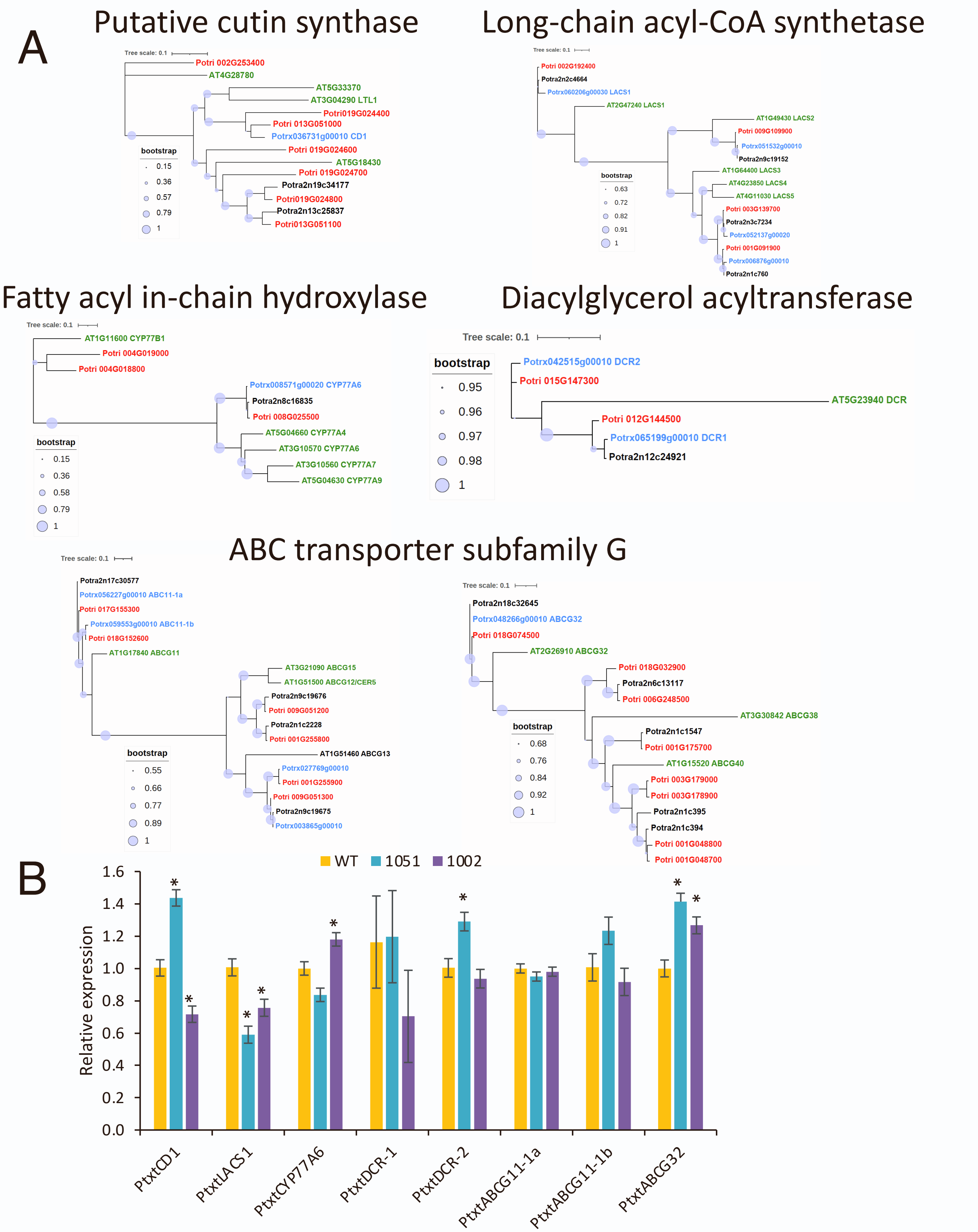

Figure S6. Genes related to cutin/wax biosynthesis and their expression in transgenic lines overexpressing *PtxtPL1-27* relative to wild type (WT). (A) Phylogenetic trees showing cutin/wax biosynthesis - related genes in *Arabidopsis thaliana* and their closest homologs in *Populus trichocarpa*, *Populus tremula* and *Populus tremula x tremuloides,* clone T89. **(B)** Expression of hybrid aspen cuticle-related genes in the leaves of transgenic lines relative to expression in WT. Mean ± SE, N=3 biological replicates. Means with asterisk represents values significantly different from WT (Post ANOVA Dunnett's test, P≤0.05).

Table S1. Net photosynthesis rate, stomatal conductance, intercellular CO_2 concentration and transpiration rate in mature leaves of wild type (WT) and *Ptxt*PL1-27 overexpressing lines 1051 and 1002. Leaves from both wild type and transgenic plants were pre-exposed for 20 min to light at 500 mmole m^{-2}s-1, prior to CO_2 assimilation monitored by an infrared gas analyser (LI-COR, LI-6400 portable photosynthetic system). Data are means ± SD, N=6.

Line	Net photosynthesis rate μmol CO_2 m^{-2} s^{-1}	P	Stomatal conductance mol H_2O m^{-2} s^{-1}	P	Intercellular CO_2 concentration μmol CO2 mol air-1	P	Transpiration rate mol H_2O m^{-2} s^{-1}	P
**WT**	13.08 (± 0.22)		0.29 (± 0.007)		345.84 (± 11.13)		8.77 (± 0.66)	
**1051**	14.58 (± 0.66)	0.0056	0.34 (± 0.013)	≤0.0001	363.98 (± 11.52)	0.0302	10.09 (± 0.23)	0.0012
**1002**	18.28 (± 1.07)	≤0.0001	0.56 (± 0.013)	≤0.0001	389.74 (± 12.23)	≤0.0001	13.33 (± 0.60)	≤0.0001

Table S3. Glycosyl linkages assignment to specific polysaccharides according to Pettolino et al.[S1] and Chen et al.[S2].

Polysaccharides	Glycosyl linkages
HM	2-, 4-, 2,4-, 4,6-, 2,3,6-Man*p*; 4-, 4,6-Glc*p*; 2-, t-Gal*p*
XGs	2-, t-Xyl*p*; 4-, 4,6-Glc*p*; 2-, t-Gal*p*; t-Fuc*p*
HG	4-, t-Gal*p*A
RG-I	2-, 2,4-, 2,3,4-Rha*p*; 4-Gal*p*A
RG-II	5-Api*f*; 3-Rha*p*; 3,4-Gal*p*A; 2-Glc*p*A
HXs	4-, 2,4-, 3,4-, t-Xyl*p*; 4-, 2,4-, t-Glc*p*A; t-Ara*f*
Arabinan	2-, 3-, 5-, 2,5-, 3,5-, t-Ara*f* or 5-, 2,5-, 3,5-Ara*p*
Type I Arabinogalactans (AG-I)	4-, 3,4-, 4,6-, t-Gal*p*; t-Ara*f*
Type II Arabinogalactans (AG-II)	2-, 3-, 6-, 3,6-, 3,4,6-, t-Gal*p*; t-Ara*f*; t-Ara*p*; t-Rha*p*
Cellulose	4-Glc*p*
Other[1]	2-, 3-, 6-, 2,4-, 2,6-, 3,4-, 3,6-, t-Glc*p*; 4-, 2,3-Rha*p*; 2-Gal*p*A; 2,3,4-Ara*p*; 2,4-Fuc*p*

Table S6. Cuticular wax compounds identified in wild-type hybrid aspen leaves. Compound specific m/z fragments and typical m/z fragmentation pattern for each wax class are shown.

Peak #	Chain length	Average coverage (mg/cm^2)	SE	RT	[M+]	[M-15]	[M-18]
Fatty acids:							
14	20	0.018	*0.003*	13.22[a]	384	369	
26	22	0.025	*0.003*	15.81[a]	412	397	
33	24	0.022	*0.003*	18.41[a]	440	425	
39	26	0.043	*0.008*	20.93[a]	468	453	
49	28	0.149	*0.018*	23.36[a]	496	481	
Aldehydes:							
12	22	0.005	*0.001*	12.91[a]	*324**		306
25	24	0.035	*0.004*	15.57[a]	352		334
32	26	0.195	*0.021*	18.26[a]	380		362
37	26			20.05[a]	*380**		362
38	28	0.268	*0.056*	20.86[a]	*408**		390
46	28			22.54[a]			390
Alkanes:							
18	25	0.068	*0.008*	14.06[a]	352		
30	27	0.241	*0.022*	16.70[a]	380		
35	29	0.008	*0.002*	19.27[a]	*408**		
Alcohols:							
10	20	0.004	*0.001*	12.20[a]	*370**	355	
15	21[c]	0.002	*0.001*	13.46[a]	*384**	369	
22	22	0.554	*0.076*	14.84[a]	*398**	383	
28	23[c]	0.003	*0.000*	16.05[a]	*411**	397	
31	24	0.274	*0.029*	17.39[a]	*426**	411	
34	25[c]	0.010	*0.002*	18.63[a]	*440**	425	
36	26	0.338	*0.027*	19.95[a]	*455**	439	
45	28	0.489	*0.070*	22.42[a]	*483**	467	
52	30	0.022	*0.004*	24.68[a]	*511**	495	
Alkyl esters:							
59	38	0.411	*0.106*	32.08[a]	564		
60	40	0.434	*0.110*	35.79[b]	592		
61	42	0.470	*0.097*	40.95[b]	620		
62	44	0.128	*0.022*	47.71[b]	648		
63	46	0.055	*0.009*	57.18[b]	676		

[a] Identified by KI and by the mass spectrum
[b] Identified only by the mass spectrum
[c] Quantifiable waxes not included in the bar graph
Grey italics indicate very small or absent m/z fragment
* Typical fragments for TMS (trimethylsilyl fragments)

Table S7. Details on primary and secondary antibodies used for immunolocalization.

Antibody	Host	Epitope	Dilution	Source
CCRC M1	mouse	Fucosylated xyloglucan	10x	Carbosource
JIM5	rat	Acidic HG	10x	University of Leeds LIBA
Anti-cutinsomes	mouse	cutinsomes	50x	Gift from Antonio Heredia
EM Goat anti-Rat IgG (H&L): 10 nm Gold	goat		50x	BBInternational
Goat anti-Mouse IgG: 2 and 10 nm Gold	goat		50x	BBInternational

Supplemental references:

S1. Pettolino FA, Walsh C, Fincher GB, Bacic A. Determining the polysaccharide composition of plant cell walls. 2012. Nat Protoc. 7: 1590–607. doi: 10.1038/nprot.2012.081.
S2. Chen D, Harris PJ, Sims IM, Zujovic Z, Melton LD. 2017. Polysaccharide compositions of collenchyma cell walls from celery (*Apium graveolens* L.) petioles. BMC Plant Biol. 17:104. doi: 10.1186/s12870-017-1046-y.
